# Synthesis, Characterization and Assessment of the Antioxidant Activity of Cu(II), Zn(II) and Cd(II) Complexes Derived from Scorpionate Ligands

**DOI:** 10.3390/molecules25225298

**Published:** 2020-11-13

**Authors:** Aurel Tăbăcaru, Rais Ahmad Khan, Giulio Lupidi, Claudio Pettinari

**Affiliations:** 1 Department of Chemistry, Physics and Environment, Faculty of Sciences and Environment, “Dunarea de Jos” University of Galati, 111 Domneasca Street, 800201 Galati, Romania; 2 Department of Chemistry, College of Science, King Saud University, P.O. Box 2455, Riyadh 11451, Saudi Arabia; raischem@gmail.com; 3 School of Pharmacy, University of Camerino, Via S. Agostino 1, 62032 Camerino, Italy; giulio.lupidi@unicam.it; 4 Istituto di Chimica dei Composti Organometallici (ICCOM-CNR), Via Madonna del Piano 10, 50019 Sesto Fiorentino, Italy

**Keywords:** scorpionate complexes, copper, zinc, cadmium, antioxidant activity

## Abstract

Seeking to enrich the yet less explored field of scorpionate complexes bearing antioxidant properties, we, here, report on the synthesis, characterization and assessment of the antioxidant activity of new complexes derived from three scorpionate ligands. The interaction between the scorpionate ligands thallium(I) hydrotris(5-methyl-indazolyl)borate (TlTp^4Bo,5Me^), thallium(I) hydrotris(4,5-dihydro-2H-benzo[g]indazolyl)borate (TlTp^a^) and potassium hydrotris(3-*tert*-butyl- pyrazolyl)borate (KTp*^t^*^Bu^), and metal(II) chlorides, in dichloromethane at room temperature, produced a new family of complexes having the stoichiometric formula [M(Tp^4Bo,5Me^)_2_] (M = Cu, **1**; Zn, **4**; Cd, **7**), [M(Tp^a^)_2_] (M = Cu, **2**; Zn, **5**; Cd, **8**), [Cu(Hpz*^t^*^Bu^)_3_Cl_2_] (**3**), [Zn(Tp^tBu^)Cl] (**6**) and [Cd(Bp*^t^*^Bu^)(Hpz*^t^*^Bu^)Cl] (**9**). The obtained metal complexes were characterized by Fourier transform infrared spectroscopy, proton nuclear magnetic resonance and elemental analysis, highlighting the total and partial hydrolysis of the scorpionate ligand Tp*^t^*^Bu^ during the synthesis of the Cu(II) complex **3** and the Cd(II) complex **9**, respectively. An assessment of the antioxidant activity of the obtained metal complexes was performed through both enzymatic and non-enzymatic assays against 1,1-diphenyl-2-picryl- hydrazyl (DPPH**^·^**), 2,2′-Azino-bis(3-ethylbenzothiazoline-6-sulfonic acid) (ABTS^+**·**^), hydroxyl (HO^·^), nitric oxide (NO**^·^**), superoxide (O_2_^−^) and peroxide (OOH**^·^**) radicals. In particular, the complex [Cu(Tp^a^)_2_]⋅0.5H_2_O (**2**) exhibited significant antioxidant activity, as good and specific activity against superoxide (O_2_^−·^), (IC50 values equal to 5.6 ± 0.2 μM) and might be identified as auspicious SOD-mimics (SOD = superoxide dismutase).

## 1. Introduction

The action of a scorpion to catch and sting the prey with its pincers and tail has been the basis of inspiration for the formation of numerous metal complexes with the particular class of nitrogen-donor chelators, i.e., poly(pyrazolyl)borates and the neutral counterpart poly(azolyl)alkanes, initially introduced by Trofimenko more than fifty years ago [1,2]. For their coordinative behavior towards metal ions, mimicking the action of a scorpion when approaching the prey, such chelators have received the designation of “scorpionate ligands” to describe, more precisely, the interchange between the bidentate and tridentate coordination modes of these species.

Scorpionate ligands have become extremely versatile due to the possibility of attaining different sterical and electronic effects by simply changing the nature, number and position of substituents on the pyrazolic rings in order to finely tune the reactivity of the metal centers. Their versatility also resides in the extensive use of both the number of heterocyclic rings and substituents to prepare complexes of main group and transition metals, thus being particularly useful for the synthesis of monomeric derivatives in which the coordination sphere of the metal ion can be rigorously controlled [3,4,5,6].

From the applicative perspective, many researches on scorpionate ligands have enriched several areas of chemistry with interesting achievements in, i.e., homogeneous catalysis, organic transformations, metalloenzyme modelling, perspective materials, and even as “spectator ligands” which do not interfere with the reaction scenarios taking place at the metal centers [6]. On the other hand, the great majority of scorpionate complexes have been the subject of deep investigations for potential applications, especially in the field of catalysis [7,8,9,10,11], bioinorganics [12,13,14] and medicine [15,16].

There are many physiological and pathological processes in living organisms like plants and animals, where reactive species are forming radicals, such as in normal cellular metabolism and in the process of senescence of the tissues. Radical stress conditions or radical pathologies are determined by an imbalance of the relationship between oxidizing and antioxidant factors, at the local level or generalized to a tissue or to one or more organs. By escaping the control of protection systems, the radicals try to rebalance their electronic structure by capturing electrons from other species to damage especially polyunsaturated fatty acids, lipids, proteins or nucleic acids, which in turn goes into free radicals formation and triggers uncontrolled radicals reactions. Oxygen, in particular, essential for most of the processes of cells, can be highly toxic. Reactive oxygen species (ROS) are potentially capable of damaging cellular components, compromising their membership operation. The uncontrolled oxidation of the lipids making up the membranes (lipoperoxidation) is the most important expression of toxicity of oxygen, the cause of functional and structural alterations found, for example, in inflammation, post-ischemic reoxygenation, intoxication by synthetic substances (foreign body) and, in some cases, leading to carcinogenesis. Endogenous protection against free radicals must be done essentially to the intervention of protective enzymes, such as glutathione peroxidase, superoxide dismutase, catalase and lactoperoxidase, both present intracellular and extracellular. Radical reactions can also be inhibited by some substances which react with the radicals, blocking the series of chain reactions that can be triggered or slowed down through the formation of more stable radicals. An inhibitor that decreases the concentration of free radicals present is said “Radical scavenger.” Biological antioxidants are natural molecules that can prevent the uncontrolled formation of free radicals and ROS, or inhibit their reaction with the biological structures. In fact, they can act by inhibiting pro-oxidant enzymes and chelating transition metal ions, which lead to the formation of radicals (preventive antioxidants), or they can interfere in the propagation phase of the chain reaction by neutralizing the radicals that are formed in that stage (chain-breaking antioxidants). In last few decades, it is now well established that oxidative stress plays a significant role in various human diseases. Prolonged exposure to oxidative stress leads to several chronic diseases, like cancer, diabetes, Parkinson’s and Alzheimer’s. From the past few decades, cisplatin and their analogous for the treatment of certain types of cancer has developed a field of research as metallodrugs (metal-based drugs) [17,18,19].

**Scheme 1:**
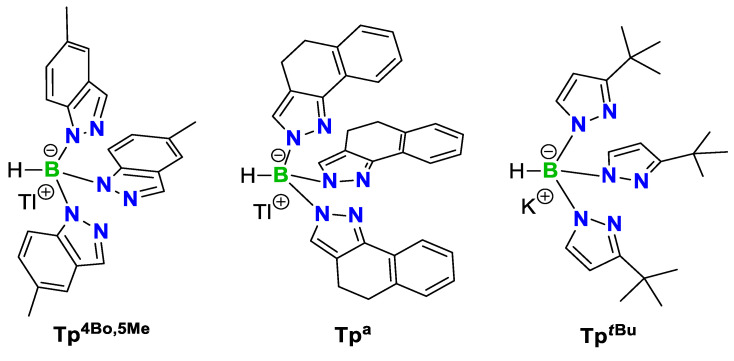
Structure of the three scorpionate ligands used in this work.

In this context, only few papers have been observed on the antioxidant capacity of scorpionate complexes with, i.e., copper(I) [20,21,22], copper(II) [23,24], zinc(II) [23,24], manganese(II) [23,24] and nickel(II) [24]. Aiming at expanding the library of scorpionate complexes with antioxidant properties, here we introduce the less explored scorpionate ligands thallium(I) hydrotris(5-methyl-indazolyl)borate (TlTp^4Bo,5Me^), thallium(I) hydrotris(4,5-dihydro-2H-benzo[g]- indazolyl)borate and potassium hydrotris(3-*tert*-butyl-pyrazolyl)borate (KTp*^t^*^Bu^) (Scheme 1) into the preparation of a new series of copper(II), zinc(II) and cadmium(II) complexes. The results regarding the synthesis, analytical and structural characterization, and antioxidant activity of the obtained metal complexes are presented.

## 2. Results and Discussion

### 2.1. Synthesis and Characterization

**Scheme 2:**
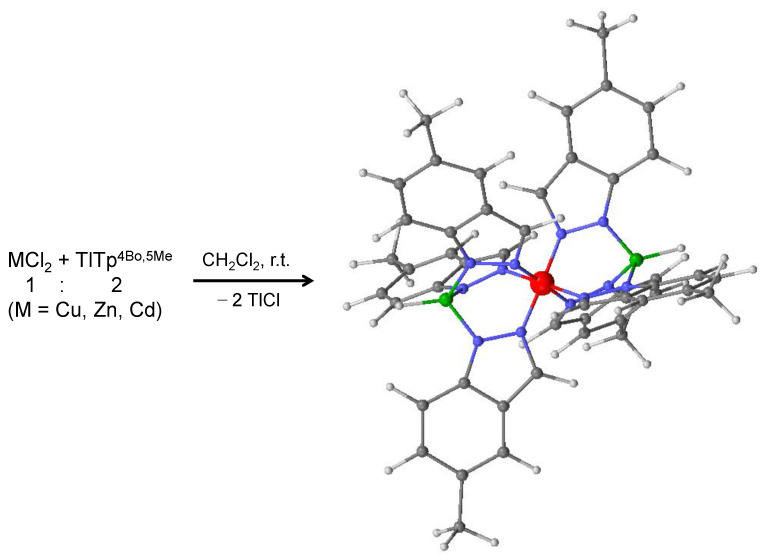
Synthesis of complexes **1**, **4** and **7**. The structure of these complexes are represented as 3D optimized models. Atoms color code: M, red; C, grey; H, light grey; N, blue; boron, green.

**Scheme 3:**
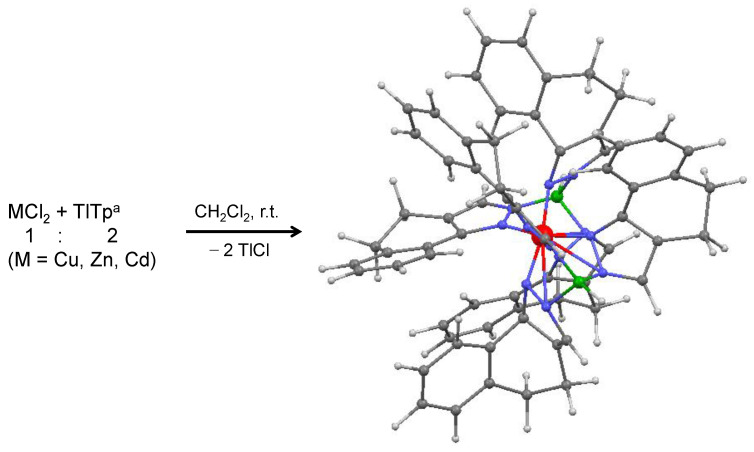
Synthesis of complexes **2**, **5** and **8**. The structure of these complexes are represented as 3D optimized models. Atoms color code: M, red; C, grey; H, light grey; N, blue; boron, green.

The syntheses of complexes **1**, **2**, **4**, **5**, **7** and **8** were conducted in dichlorometane at room temperature for two hours, from the reaction between MCl_2_ (M = Cu, Zn, Cd) and the scorpionate ligands Tp^4Bo,5Me^ and Tp^a^, in the 1:2 molar ratio (Scheme 2 and Scheme 3). They were obtained in the form of pure powders which are air and moisture stable, present good solubility in most organic solvents and are insoluble in water. The desolvated form of complex **4**, [Zn(Tp^4Bo,5Me^)_2_], had already been reported by Rheingold et al., who obtained it by the reaction of ZnCl_2_ and TlTp^4Bo,5Me^ in the 1:2 ratio, in a 1:1 mixture of tetrahydrofuran (THF) and dichloromethane, under stirring for 2–3 h, and with a different work up to isolate the final pure product [25]. Similarly, the desolvated form of complex **5**, [Zn(Tp^a^)_2_], had already been reported by Rheingold et al., who obtained it by the dropwise addition of 0.5 equivalent of a THF solution of ZnCl_2_ to a stirred dichloromethane solution of TlTp^a^, and with a different work up to isolate the final pure product [26]. Elemental analyses, along with the integration of the peaks observed in the ^1^H NMR spectra, suggested that the stoichiometric formulation metal:ligand is 1:2 in all these complexes as two scorpions attack the same prey simultaneously. Although the scorpionate ligands Tp^4Bo,5Me^ and Tp^a^ are bulkier and more sterically hindering with respect to the parent ligands tris(pyrazolyl)hydroborate (Tp) and tris(3,5-dimethylpyrazolyl)hydroborate (Tp*), their coordination to the three metal ions in the 1:2 ratio was, however, possible due to maggior cone and wedge angles that these two types of ligands possess. In these complexes the metal ion is coordinated by two ligands which employ three nitrogen atoms from the two-position of pyrazolyl cycles, in a tridentate fashion, thus giving the metal ion an octahedral geometry. Such coordinative aptitudes have already been observed in the 1:2 complexes [M(Tp)_2_] (M = Cu [27], Zn [28], Cd [29]) and [M(Tp*)_2_] (M = Cu [30], Zn [31], Cd [32]). The progressive increase of sterical hindering of Tp^x^ scorpionate ligands was found to lead, in the corresponding complexes, to a deepening of the hydrophobic pocket around the metal ions [6].

In the IR spectra of complexes **1**, **2**, **4**, **5**, **7** and **8**, the characteristic band for the stretching vibration of B−H bond is observed in the region 2450–2530 cm^−1^, and the corresponding wavenumber values are shifted to higher frequencies with respect to those of the free ligands Tp^4Bo,5Me^ and Tp^a^ (Figure S1, Supplementary Information) [33]. In the region 2800–3000 cm^−1^ there are signals of weak or medium intensity, which are assigned to the stretching of methyl C−H bonds from Tp^4Bo,5Me^ and ethylenic –CH_2_CH_2_− bonds (tethering group) from Tp^a^. Over 3000 cm^−1^ the observed weak absorptions are assigned to the vibration of aromatic C−H bonds. In the region 1500–1650 cm^−1^ the observed medium or strong absorptions are characteristic for the so-called “breathing” of the pyrazolyl rings [34], which also undergoes significant changes in terms of intensity and frequency with respect to the free ligands, as a sign of successful coordination to the metal ions. ^1^H NMR spectra of the complexes containing the diamagnetic zinc(II) and cadmium(II) ions (**4**, **5**, **7** and **8**) present a unique set of signals that are shifted towards the higher field with respect to the free ligands, also suggesting the equivalence of the three pyrazolyl rings at room temperature. The shift towards higher field could be assigned to the ring current effect of aromatic cycles interpenetrating within an octahedral structure [26], whereas the equivalence of pyrazolyl rings could be explained by the rapid movement involving the pyrazolyl moieties bonded to metal centers [35]. Interestingly, the observed chemical shifts of complex **5** are considerably different from those of the desolvated form [26]. A possible reason for such a difference may reside in the fact that complex **5** undergoes partial dissociation in dichloromethane solution, and therefore, the chemical shifts are average values between those of the dissociated complex and ligand. Moreover, our synthesized complex has four dichloromethane molecules which could make a sort of electrostatic interaction with the complex.

**Scheme 4:**
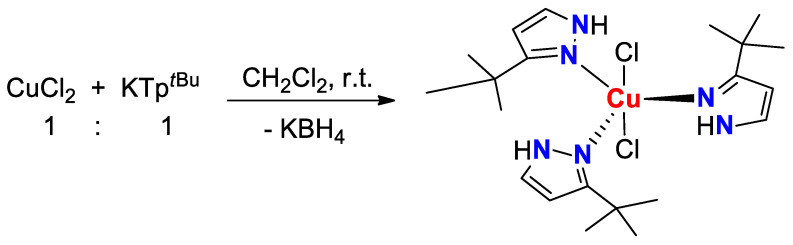
Synthesis of complex **3**.

In the case of Tp^tBu^, which is much more hindering than the other ligands, possessing a cone angle of 251° and a wedge angle of 29°, the coordinative behavior towards Cu(II), Zn(II) and Cd(II) ions surprisingly changes. As such, in the reaction of CuCl_2_ with KTp^tBu^ in dichloromethane at room temperature, either using 1:1 or 1:2 metal:ligand ratio, the ligand loses its initial identity of a scorpionate-type one, completely hydrolyzing through cleavage of B−N bonds and releasing the three neutral 3-tert-butylpyrazole molecules which, however, coordinate to the Cu(II) center to finally afford complex **3** with trigonal-bipyramidal geometry (Scheme 4). The presence of neutral pyrazole in this complex was denounced by the missing band characteristic of the stretching vibration of B−H bond, and by the appearance, instead, of a broad band at 3148 cm^−1^ due to the vibration of the pyrazolyl N−H bond. This relatively rare behavior was already observed with other scorpionate ligands in the presence of transition metal(II) or metal(III) ions [36,37,38,39,40,41,42,43], and it is due not only to the different acidity of the metal centers, but also by the degree of steric crowding around the boron center in the corresponding scorpionate ligands [44].

**Scheme 5:**
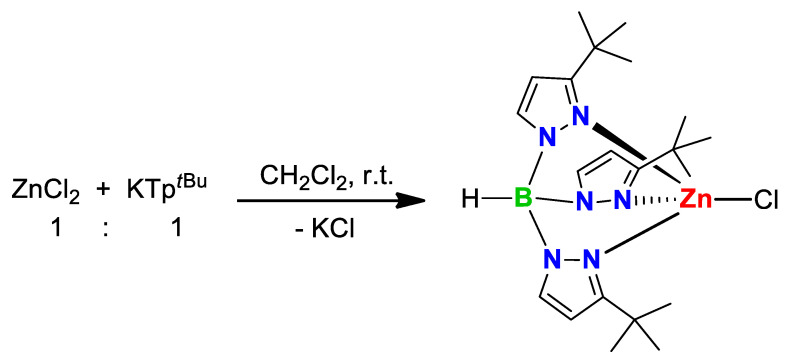
Synthesis of complex **6**.

In the reaction of ZnCl_2_ with KTp^tBu^ in dichloromethane at room temperature, either using 1:1 or 1:2 metal:ligand ratio, the ligand maintained its scorpionate identity, thus leading to the formation of complex **6** with tetrahedral geometry (Scheme 5). Elemental analysis indicated the stoichiometric formulation metal:ligand of 1:1 in this complex. Looney et al., had already obtained the same complex by a different synthetic procedure, involving the reaction of a benzene solution of the complex precursor [(Tp^tBu^)ZnMe] with hydrochloric acid at room temperature [45]. Although using either the 1:1 or 1:2 metal:ligand ratio, it is obvious that the sterical hindering of the ligand Tp^tBu^, much higher than the other two ligands used in this work, induced by the tert-butyl group on the three-position of the pyrazolyl ring, does not allow the coordination of two ligand molecules at the metal center. For such species, the chemical shifts were found at 7.6, 6.1 and 1.43 ppm, for which the integration ratio is 1:1:9. Interestingly, an additional set of signals was also observed in the ^1^H NMR spectrum of complex 6, which are located at 7.7, 6.18 and 1.36 ppm (integration ratio 1:1:9). These signals stand for a similar species, possibly in equilibrium with the first one, in which one of the three pyrazoles is in a different environment (k^2^-*N,N* hapticity).

**Scheme 6:**
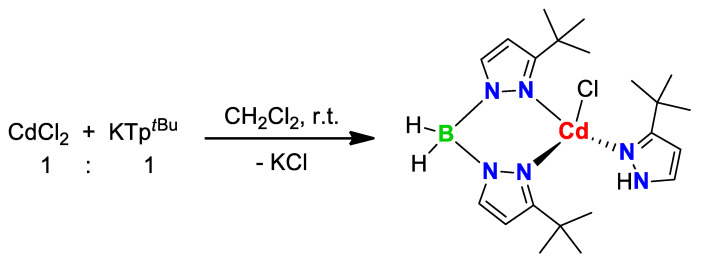
Synthesis of complex **9**.

The reaction of CdCl_2_ with KTp^tBu^ in the 1:2 ratio, in dichloromethane at room temperature, has led to partial hydrolysis of the ligand, in which only one 3-tert-butylpyrazole molecule was displaced from the boron atom, coordinating separately to the Cd(II) center (Scheme 6). Thus, through this partial hydrolysis, a restructuring process of the scorpionate ligand occurred, passing from a species with k^3^-*N*,*N*,*N* hapticity to a species with k^2^-*N,N* hapticity (Bp^tBu^) to finally give the tetrahedral complex **9**. To the best of our knowledge, no other similar behavior was reported so far in the literature. However, Reger et al. have reported the reaction of the same reagents in tetrahydrofuran at room temperature, in which the ligand maintained its structural integrity, without undergoing any hydrolysis reaction [46]. In this case, the solvent effect is perhaps the most influencing factor for the structural integrity of the scorpionate ligand Tp^tBu^. The IR spectrum showed both the absorptions specific to the BH_2_ group at 2449 and 2484 cm^−1^, and the absorption specific to the NH function of the neutral pyrazolyl cycle at 3187 cm^−1^ (Figure S9, Supplementary Information). The ^1^H NMR spectrum showed, beside the peaks specific to the coordinated ligand Bp^tBu^, one set of peaks assignable to the neutral 3-tert-butylpyrazole, the low-intensity peak at 9.83 ppm being due to the proton from the pyrazolyl NH function.

### 2.2. Antioxidant Activity

**Figure 1:**
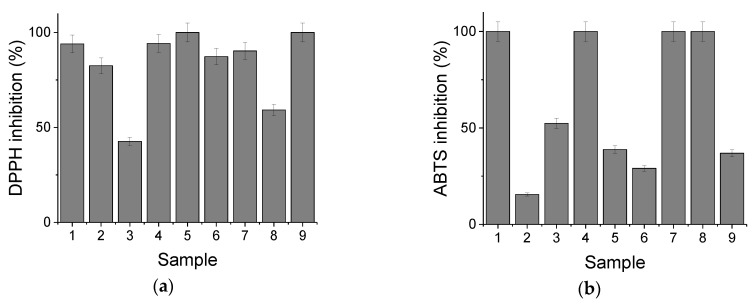
Scavenger activity of metal complexes towards 1,1-diphenyl-2-picrylhydrazyl (DPPH) (**a**) and 2,2′-Azino-bis(3-ethyl- benzothiazoline-6-sulfonic acid) (ABTS) radicals (**b**).

The antioxidant properties of the scorpionate metal complexes **1**-**9** were studied for their radical scavenging activity towards 1,1-diphenyl-2-picrylhydrazyl (DPPH**^·^**), 2,2′-Azino-bis(3-ethyl- benzothiazoline-6-sulfonic acid) (ABTS^+^), hydroxyl (HO), nitric oxide (NO), superoxide (O_2_^−^) and peroxide (OOH**^·^**) radicals by using various standard assays as mentioned in the experimental section. The activity of scavengers is demonstrated against both DPPH**^·^** and ABTS^+^ radicals (Figure 1), and the values of the respective IC_50_ compared to the calculated value for Trolox (standard) are shown in Table 1. Antioxidant activity against DPPH**^·^** was exhibited by the copper complex [CuCl_2_(Hpz*^t^*^Bu^)_3_] (**3**) and cadmium complex [Cd(Tp^a^)_2_]⋅1.5CH_2_Cl_2_ (**8**), which is comparable to that of the already reported series of [M(Tm)(diimine)](ClO_4_) complexes (M = Mn(II), Ni(II), Cu(II) or Zn(II); Tm = hydrotris(methimazolyl)borate; diimine = 2,2′-bipyridyl or 1,10-phenantroline) [24]. ABTS^+**·**^ scavenging was observed in the copper complexes [Cu(Tp^a^)_2_]⋅0.5H_2_O (**2**) and [CuCl_2_(Hpz*^t^*^Bu^)_3_] (**3**), zinc complexes [Zn(Tp^a^)_2_]⋅4CH_2_Cl_2_ (**5**) and [Zn(Tp*^t^*^Bu^)Cl] (**6**) and cadmium complex [Cd(Bp*^t^*^Bu^)(Hpz*^t^*^Bu^)Cl] (**9**), following concentration dependent patterns.

From Table 1 it can be observed that the obtained complexes show lower antioxidant activity with respect to Trolox, used as control. As described in the methods and reported in Table 1, complexes **1**–**9** were also analyzed for the scavenger activities against radicals such as O_2_**^−^**, OH**^·^**, HOO and NO. The results for the tested complexes show that the Cu(II) complex **2** reacts specifically with superoxide anion (O_2_**^−^**) and could be considered as a good SOD-mimics, with a IC_50_ value equal to 5.6 ± 0.2 μM, which is about 12 times less than that reported for native bovine Cu-Zn-SOD (IC_50_ = 0.48 μM) [47]. The SOD activity of complex **2** was also found comparable to other reported scorpionate complexes, such as [Cu(PPh_2_(4-C_6_H_4_COOH))(Tp^4Br^)] (PPh_2_(4-C_6_H_4_COOH) = 4-(diphenylphosphane)benzoic acid, Tp^4Br^ = hydrotris(4-bromo-1*H*-pyrazol-1-yl)borate) with IC_50_ = 6.2 µM [20], and [Cu(PPh_2_(4-C_6_H_4_COOH))_2_(pzTp)] [pzTp = tetrakis(1*H*-pyrazol-1-yl)borate] with IC_50_ = 4.6 µM [21]. Complex **3** also shows lower SOD mimics activity with respect to complex **2** (47 times less, and 500 times less with respect to native bovine Cu-Zn-SOD). However, the other complexes do not show any activity towards the radicals O_2_**^−^**, OH, OOH and NO**^·^**. By examining the data reported in Table 1, the results suggest that in the series of Cu(II) complexes, by replacement with less bulky ligands, the scavenger activity against superoxide anion is retained, as reported for the enzyme Cu/Zn superoxide dismutase used by the biological systems to decrease the damage caused by the same radical.

The scavenger activity against synthetic radicals, such as DPPH and, above all, ABTS^+**·**^, seems to be more marked along the complexes in the following order: Cu^2+^ > Zn^2+^ > Cd^2+^. Overall, the results show that, from all the synthesized scorpionate complexes **1**–**9**, the complex [Cu(Tp^a^)_2_]⋅0.5H_2_O (**2**) exhibits a selective good antioxidant activity (10 times less than Trolox, used as control) towards ABTS^+^ radical, and can act as a good SOD mimics with preferential selectivity against superoxide radical.

## 3. Materials and Methods

### 3.1. General

All the chemicals and reagents were purchased from Sigma-Aldrich (Darmstadt, Germany) and used as received, without further purification. All the solvents were distilled prior to use. The scorpionate ligands thallium(I) hydrotris(5-methyl-indazolyl)borate (TlTp^4Bo,5Me^) [25], thallium(I) hydrotris(4,5-dihydro-2H-benzo[g]indazolyl)borate (TlTp^a^) [26], and potassium hydrotris(3-*tert*-butyl-pyrazolyl)borate (KTp*^t^*^Bu^) [48] were prepared by the literature methods. The IR spectra were recorded from 4000 to 650 cm^−1^ with a Perkin Elmer Spectrum 100 instrument (Perkin-Elmer, Shelton, CT, USA) by attenuated total reflectance (ATR) on a CdSe crystal. The ^1^H NMR spectra were acquired at room temperature with a VXR-300 Varian spectrometer (Varian Inc., Palo Alto, CA, USA) operating at 400 MHz, using tetramethylsilane as the internal standard. Elemental analyses (N, C, H) were performed with a Fisons Instruments 1108 CHNS-O Elemental Analyzer (Thermo Scientific, Waltham, MA, USA). Before performing the analytical characterization, all the samples were dried in vacuo (50 °C, ~0.1 Torr) until a constant weight was reached. Melting points were measured with a Stuart SMP3 instrument (Keison Products, Essex, UK) equipped with a capillary apparatus.

### 3.2. Syntheses of Scorpionate Complexes

[Cu(Tp^4Bo,5Me^)_2_]⋅0.5CH_2_Cl_2_ (**1**). In 20 mL of dichloromethane, 0.1219 g of TlTp^4Bo,5Me^ (0.2 mmol) were dissolved, and then 0.0171 g of CuCl_2_∙2H_2_O (0.1 mmol) were added. After several minutes, a white precipitate was formed and identified as thallium(I) chloride and, after 2 h of stirring at room temperature, it was filtered off. The filtrate was evaporated, and a dark green solid was isolated, which was dried in vacuo at 35 °C. Complex **1** is soluble in chlorinated solvents, alcohols, acetonitrile, dimethylsulfoxide and dimethylformamide, and is insoluble in water. Yield: 85%. Mp: 268–272 °C. *Anal.* Calc. for C_48_H_44_B_2_CuN_12_ (0.5CH_2_Cl_2_, FW = 916.57 g mol^−1^): N, 18.34; C, 63.55; and H, 4.95%. Found: N, 18.16; C, 63.76; and H, 5.31%. IR *ν*(cm^−1^): 3027 (w), 2970 (w), 2918 (w), 2860 (w), 2476 (m) ν(B-H), 1630 (m), 1511 (s) ν(C=C + C=N), 1438 (m), 1340 (s), 1264 (s), 1155 (s), 1114 (vs), 1000 (s), 937 (m), 823 (s) and 697 (s).

[Cu(Tp^a^)_2_]⋅0.5H_2_O (**2**). In 20 mL of dichloromethane, 0.1447 g of TlTp^a^ (0.2 mmol) were dissolved, and then 0.0171 g di CuCl_2_∙2H_2_O (0.2 mmol) were added. After several minutes, a white precipitate was formed and identified as thallium(I) chloride and, after 2 h of stirring at room temperature, it was filtered off. The filtrate was evaporated, and a dark green solid was isolated, which was dried in vacuo at 35 °C. Complex **2** is soluble in chlorinated solvents, alcohols, acetonitrile, dimethylsulfoxide and dimethylformamide, and is insoluble in water. Yield: 80%. Mp: 145-148 °C. *Anal.* Calc. for C_66_H_56_B_2_CuN_12_ (0.5H_2_O, FW = 1111.41 g mol^−1^): N, 15.12; C, 71.33; and H, 5.17%. Found: N, 14.96; C, 70.96; and H, 5.14%. IR *ν*(cm^−1^): 3055 (w), 2930 (w), 2840 (w), 2454 (m) ν(B-H), 1581 (w), 1557 (w), 1470 (s) ν(C=C + C=N), 1436 (m), 1396 (m), 1129 (vs), 1098 (s), 1002 (m) and 767 (s).

[Cu(Hpz*^t^*^Bu^)_3_Cl_2_] (**3**). In 20 mL of dichloromethane, 0.117 g of KTp*^t^*^Bu^ (0.2 mmol) were dissolved, and then 0.0342 g of CuCl_2_∙2H_2_O (0.2 mmol) were added. After several minutes, a white precipitate was formed and identified as potassium tetrahydroborate and, after 2 h of stirring at room temperature, it was filtered off. The filtrate was evaporated, and a dark green solid was isolated, which was dried in vacuo at 35 °C. Complex **3** is soluble in chlorinated solvents, alcohols, acetonitrile dimethylsulfoxide and dimethylformamide, and is insoluble in water. Yield: 75%. Mp: 118-121 °C. *Anal.* Calc. for C_21_H_36_Cl_2_CuN_6_ (FW = 507.00 g mol^−1^): N, 16.58; C, 49.75; and H, 7.16%. Found: N, 16.23; C, 50.38; and H, 7.26%. IR *ν*(cm^−1^): 3148 (m br) ν(N-H_pz_), 2962 (m), 2870 (w), 1552 (m), 1483 (m) ν(C=C + C=N), 1367 (m), 1124 (vs), 990 (s), 954 (s), 791 (vs) and 728 (m).

[Zn(Tp^4Bo,5Me^)_2_]⋅CH_2_Cl_2_ (**4**). In 20 mL of dichloromethane, 0.1219 g TlTp^4Bo,5Me^ (0.2 mmol) were dissolved, and then 0.0172 g of ZnCl_2_∙2H_2_O (0.1 mmol) were added. After several minutes, a white precipitate was formed and identified as thallium(I) chloride and, after 2 h of stirring at room temperature, it was filtered off. The filtrate was evaporated, and a light yellow solid was isolated, which was dried in vacuo at 35 °C. Complex **4** is soluble in chlorinated solvents, alcohols, acetonitrile, dimethylsulfoxide and dimethylformamide, and is insoluble in water. Yield: 90%. Mp: 302-305 °C. *Anal.* Calc. for C_48_H_44_B_2_ZnN_12_ (CH_2_Cl_2_, FW = 960.88 g mol^−1^): N, 17.49; C, 61.25; and H, 4.83%. Found: N, 17.61; C, 61.65; and H, 4.71%. IR *ν*(cm^−1^): 3027 (w), 2917 (w), 2475 (w) ν(B-H), 1629 (w), 1513 (s) ν(C=C + C=N), 1438 (m), 1263 (s), 1157 (s), 1119 (vs), 1007 (vs), 937 (w), 823 (s) and 799 (s). ^1^H NMR (CD_2_Cl_2_, 293 K): δ, 8.08–8.11 d (6H, *H*-7), 7.57 s (6H, *H*-3), 7.31 d (12H, *H*-6, *H*-4) and 2.39 s (18H, C*H*_3_).

[Zn(Tp^a^)_2_]⋅4CH_2_Cl_2_ (**5**). In 20 mL of dichloromethane, 0.1447 g of TlTp^a^ (0.2 mmol) were dissolved, and then 0.0172 g of ZnCl_2_∙2H_2_O (0.1 mmol) were added. After several minutes, a white precipitate was formed and identified as thallium(I) chloride and, after 2 h of stirring at room temperature, it was filtered off. The filtrate was evaporated, and a light yellow solid was isolated, which was dried in vacuo at 35 °C. Complex **5** is soluble in chlorinated solvents, alcohols, acetonitrile, dimethylsulfoxide and dimethylformamide, and is insoluble in water. Yield: 88%. Mp: 225–228 °C. *Anal.* Calc. for C_66_H_56_B_2_ZnN_12_ (4CH_2_Cl_2_, FW = 1443.96 g mol^−1^): N, 11.64; C, 58.23; and H, 4.47%. Found: N, 11.27; C, 57.71; and H, 4.30%. IR *ν*(cm^−1^): 3057 (w), 2933 (w), 2837 (w), 2467 (m) ν(B-H), 1605 (m), 1557 (m) ν(C=C + C=N), 1471 (s), 1380 (s), 1128 (vs), 1100 (s), 1004 (s) and 770 (s). ^1^H NMR (CD_2_Cl_2_, 293 K): δ, 8.07–8.09 d (6H, *H*-6′), 7.62 s (6H, *H*-5), 7.25–7.33 m (18H, *H*-3′,4′,5′), 2.87–2.89 m (12H, C*H*_2_) and 2.71–2.73 m (12H, C*H*_2_).

[Zn(Tp*^t^*^Bu^)Cl] (**6**). In 20 mL of dichloromethane, 0.058 g of KTp*^t^*^Bu^ (0.1 mmol) were dissolved, and then 0.0172 g of ZnCl_2_∙2H_2_O (0.1 mmol) were added. After several minutes, a white precipitate was formed and identified as potassium chloride and, after 2 h of stirring at room temperature, it was filtered off. The filtrate was evaporated, and a light yellow solid was isolated, which was dried in vacuo at 35 °C. Complex **6** is soluble in chlorinated solvents, alcohols, acetonitrile, dimethylsulfoxide and dimethylformamide, and is insoluble in water. Yield: 82%. Mp: 245–248 °C. *Anal.* Calc. for C_21_H_34_ClZnBN_6_ (FW = 482.18 g mol^−1^): N, 17.43; C, 52.31; and H, 7.11%. Found: N, 17.10; C, 52.72; and H, 7.36%. IR *ν*(cm^−1^): 2957 (m), 2868 (w), 2503 (w) ν(B-H), 1560 (w), 1501 (s) ν(C=C + C=N), 1463 (w), 1364 (m), 1345 (m), 1260 (m), 1195 (s), 1168 (s), 1123 (vs) and 786 (s). ^1^H NMR (CD_2_Cl_2_, 293 K): δ, 1.43 s (27H, C*H*_3_), 6.1 d (3H, 4-C*H*_Tp_) and 7.6 d (3H, 5-C*H*_Tp_).

[Cd(Tp^4Bo,5Me^)_2_] (**7**). In 20 mL of dichloromethane, 0.1219 g of TlTp^4Bo,5Me^ (0.2 mmol) were dissolved, and then 0.0183 g of CdCl_2_ (0.1 mmol) were added. After several minutes, a white precipitate was formed and identified as thallium(I) chloride and, after 2 h of stirring at room temperature, it was filtered off. The filtrate was evaporated, and a light yellow solid was isolated, which was dried in vacuo at 35 °C. Complex **7** is soluble in chlorinated solvents, alcohols, acetonitrile, dimethylsulfoxide and dimethylformamide, and is insoluble in water. Yield: 92%. Mp: 305-310 °C. *Anal.* Calc. for C_48_H_44_B_2_CdN_12_ (FW = 922.98 g mol^−1^): N, 18.21; C, 62.46; and H, 4.80%. Found: N, 18.00; C, 61.93; and H, 4.80%. IR *ν*(cm^−1^): 3026 (w), 2918 (w), 2860 (w), 2477 (m) ν(B-H), 1629 (m), 1512 (s) ν(C=C + C=N), 1437 (m), 1361 (m), 1334 (s), 1263 (s), 1155 (s), 1115 (vs), 1005 (vs), 937 (m), 822 (s), 699 (s). ^1^H NMR (CD_2_Cl_2_, 293 K): δ, 8.15-8.13 d (6H, *H*-7), 7.84 s (6H, *H*-3), 7.39 s (6H, *H*-6), 7.35–7.33 d (6H, *H*-4) and 2.42 s (18H, C*H*_3_).

[Cd(Tp^a^)_2_]⋅1.5CH_2_Cl_2_ (**8**). In 20 mL of dichloromethane, 0.1447 g of TlTp^a^ (0.2 mmol) were dissolved, and then 0.0183 g of CdCl_2_ (0.1 mmol) were added. After several minutes, a white precipitate was formed and identified as thallium(I) chloride and, after 2 h of stirring at room temperature, it was filtered off. The filtrate was evaporated, and a light yellow solid was isolated, which was dried in vacuo at 35 °C. Complex **8** is soluble in chlorinated solvents, alcohols, acetonitrile, dimethylsulfoxide and dimethylformamide, and is insoluble in water. Yield: 85%. Mp: 300-305 °C. *Anal.* Calc. for C_66_H_56_B_2_CdN_12_ (1.5CH_2_Cl_2_, FW = 1278.66 g mol^−1^): N, 13.14; C, 63.40; and H, 4.65%. Found: N, 12.88; C, 62.93; and H, 4.58%. IR *ν*(cm^−1^): 3052 (w), 2934 (m), 2841 (w), 2451 (m) ν(B-H), 1581 (w), 1558 (m), 1472 (m) ν(C=C + C=N), 1393 (s), 1145 (vs), 1100 (s), 1004 (s) and 895 (m). ^1^H NMR (CD_2_Cl_2_, 293 K): δ, 7.62 s (6H, *H*-6′), 7.32-7.30 d (6H, *H*-5), 6.81–6.79 d, 6.70–6.66 t, 6.08–6.04 t (18H, *H*-3′,4′,5′), 2.38-2.34 t (12H, C*H*_2_) and 2.03–1.99 t (12H, C*H*_2_).

[Cd(Bp*^t^*^Bu^)(Hpz*^t^*^Bu^)Cl] (**9**). In 20 mL of dichloromethane, 0.116 g of KTp*^t^*^Bu^ (0.2 mmol) were dissolved, and then 0.0183 g of CdCl_2_ (0.1 mmol) were added. After several minutes, a white precipitate was formed and identified as potassium chloride and, after 2 h of stirring at room temperature, it was filtered off. The filtrate was evaporated, and a light yellow solid was isolated, which was dried in vacuo at 35 °C. Complex **9** is soluble in chlorinated solvents, alcohols, acetonitrile, dimethylsulfoxide and dimethylformamide, and is insoluble in water. Yield: 78%. Mp: 130–133 °C. *Anal.* Calc. for C_21_H_36_ClCdBN_6_ (FW = 531.22 g mol^−1^): N, 15.82; C, 47.48; and H, 6.83%. Found: N, 15.66; C, 47.45; and H, 6.59%. IR *ν*(cm^−1^): 3187 (w) ν(N-H_pz_), 2960 (s), 2866 (w) ν(CH_3_), 2484 (w), 2449 (w) ν(BH_2_), 1501 (s) ν(C=C + C=N), 1460 (m), 1356 (m), 1258 (m), 1197 (vs), 1166 (s), 1152 (vs) and 772 (s). ^1^H NMR (CDCl_3_, 293 K): δ, 9.83 s br (1H, N-*H*_pz_), 7.57 d (2H, *H*-5_Bp_), 7.47 s (1H, *H*-2), 6.08–6.06 d (1H, *H*-3_pz_), 6.05–6.02 d br (2H, *H*-4_Bp_) and 1.39–1.33 m (27H, C*H*_3_).

### 3.3. Antioxidant Activity Assays

We have carried out both enzymatic and non-enzymatic assays to evaluate the antioxidant properties of all the synthesized scorpionate complexes of Cu(II), Zn(II) and Cd(II) (**1**–**9**). All complexes were used at an initial concentration of 2.5 mM diluted in methanol.

#### 3.3.1. Analysis of the DPPH Radical Scavenging Activity

The antioxidant properties of the compounds or control substances have been measured by DPPH, which is able to react with various natural or synthetic antioxidants [49]. A dose response curve was plotted to determine the IC_50_ values, and the following formula was used to calculate the percentage of radical scavenging:

Radical scavenging % = [(A_0_ − A_c_)/A_0_] × 100

where A_0_ is the absorbance of the control sample, containing the reagents except the test substance, and A_c_ is the absorbance of the solution of the various complexes and of the control measured after 30 min of incubation at 37 °C. Any determination is performed in triplicate.

In order to determine the DPPH radical scavenging activity of different scorpionate complexes, the compounds or standards in methanol were diluted in series in the 96 well plate with a flat bottom. The solvent used is usually the same used to dissolve the samples. In various wells containing the compounds and standards, 200 μL of DPPH in methanol solution (100 μM) were added to reach a final volume of 250 μL in all wells. After incubation at 37 °C for 20 min, the absorbance of samples was determined at 490 nm (Multiskan JX, Thermo Labsystems Oy, Vantaa, Finland). Trolox was used as a control and was loaded into the plate with the same method and dilution of the samples.

#### 3.3.2. Analysis of ABTS Radical Scavenging Activity

Total antioxidant activity was assessed as a measure of radical scavenging activity of the complexes examined against the cationic radical ABTS^+^. The method is valid for both hydrophilic and hydrophobic antioxidants. The working solution is produced by the oxidation of an initial solution of ABTS, which is oxidized to ABTS^+**·**^ through the reaction with K_2_S_2_O_8_ (2.45 mM) and left in the dark for 12 h. After incubation, the ABTS^+**·**^ solution is diluted with methanol to obtain an absorbance of 0.706 ± 0.001 units at 734 nm, which is used as a solution for the analysis of scavenger activity.

#### 3.3.3. Measurement of the Radical Scavenging Capacity

Radical scavenging ability is measured with a modified method described initially by Re et al. [50] for the application of a microplate 96 wells. Compounds or standards in methanol were diluted in series in the 96 well plate, as previously described. Trolox was used as a control and was loaded into the plate with the same method and dilution of the samples. The reaction is started with the addition of 200 μL of the ABTS^+**·**^ solution (final volume in the wells, 250 μL). The reaction mixture is allowed to stand in the dark for 10 min at room temperature, and the absorbance at 630 nm is measured (Multiskan JX, Thermo Labsystems Oy, Vantaa, Finland). The radical scavenging activity is estimated through the absorbance decrease at 630 nm and can be expressed as the equivalent Trolox.

#### 3.3.4. Measurements of Superoxide Radicals Scavenging Activity

The scavenging activity of superoxide radicals was determined by the method of Liu and Ng [51], slightly modified by Kang and Lee [52]. Superoxide radicals are generated in the phenazine methosulfate-nicotinamide adenine dinucleotide (PMS-NAD) system, responsible for the oxidation of NADH (reduced form of NAD) that was analyzed by reducing the nitro blue tetrazolium chloride NBT in a microplate assay.

In a 96 well plate, samples or control were subjected to a serial dilution with 20 mM Tris-HCl (pH 8.0). Superoxide radicals are generated by adding in 200 μL of 20 mM Tris-HCl (pH 8.0), containing 78 mM NADH, NBT 50 mM to each well and 10 μL are added to the various samples of PMS 10 mM. The reaction between superoxide radicals and NBT develops a blue color, whose intensity decreases proportionally in the presence of the antioxidant compound. The change in absorbance in the various wells is measured at 560 nm with a microplate reader (Molecular Devices, Sunnyvale, CA, USA). L-ascorbate was used as a positive control. The interaction relationship with the superoxide radical (%) was calculated using the following formula:

superoxide radical (%) = ((A − A_1_)/A) × 100

where A is the absorbance of the positive control, and A_1_ is the absorbance of the test samples.

#### 3.3.5. Measurements of Hydrogen Peroxide Radicals Scavenging Activity

In order to determine the scavenging activity of H_2_O_2_ of different complexes or standards in methanol, the compounds were diluted in series in a 96-well flat-bottom plate with the same procedure used in previous assays. Then, 200 μL of peroxide solution was added to the various wells hydrogen in PBS (final volume, 250 μL). After 10 min of incubation, the absorbance was measured at 230 nm with a microplate reader.

#### 3.3.6. Measurements of Hydroxyl Radicals Scavenging Activity

The scavenging activity of superoxide radicals was determined by the method of Liu and Ng [51], slightly modified by Kang and Lee [52]. Hydroxyl radicals, generated in the L-ascorbic acid-CuSO_4_ system, with the reduction of Cu^2+^, were analyzed by the oxidation of cytochrome c in 96 microplate wells. The hydroxyl radicals were generated in 200 μL of 10 mM buffer sodium phosphate (pH 7.4), containing 100 mM L-ascorbic acid, 100 mM CuSO_4_, 12 μM reduced cytochrome c and the samples to be analyzed in different concentrations. The reduction of cytochrome c is produced with the addition of an excess of dithiothreitol to a solution of cytochrome c and followed by Sephadex G-15 chromatography (bed volume, 10 mL) to remove the excess of dithiothreitol. 

The compounds or standards in methanol were diluted in series in a 96-well flat-bottomed plate. Stock solution was prepared in phosphate buffer (pH 7.4) containing 100 μM L-ascorbic acid and 100 μM CuSO_4_. Then, 200 μL of this solution was added to each well and, finally, 10 μL of 120 μM reduced cytochrome c, was added to the plate and was left to incubate for 20 min at room temperature. The change in absorbance, caused by the oxidation of cytochrome c, was measured at 550 nm with a microplate reader (Molecular Devices, Sunnyvale, CA, USA). A thiourea solution (500 μM) was used as a positive control. The scavenging activity of hydroxyl radicals of 500 mg/mL solution of thiourea was taken as 100%. The scavenging activity of hydroxyl radicals has been calculated using the following formula:

hydroxyl scavenging radicals (%) = (A − A_0_)/(A_T_ − A_0_) × 100

where A is the absorbance of the samples, and A_T_ and A_0_ is the absorbance of the thiourea and the control, respectively.

#### 3.3.7. Measurements of Nitric Oxide Scavenging Activity

In order to determine the scavenging activity of the metal complexes against NO radicals, samples were added to a 96-well plate dish with a flat bottom, and were serially diluted by the same procedure used in the previous assays. In all wells, 100 μL of PBS and 50 μL of sodium nitroprusside dissolved in saline phosphate buffer (PBS), were added. The plate with the samples was incubated in the presence of light at room temperature for 150 min. Finally, an equal volume of 50 μL of Griess reagent [53] was added to each well in order to measure the nitrite content. The plate was incubated at room temperature for 10 min until chromophore formation, and the absorbance was measured at 577 nm in a microplate reader (Molecular Devices, Sunnyvale, CA, USA).

## 4. Conclusions

We have here reported the synthesis and characterization of nine Zn, Cu and Cd complexes derived from scorpionate ligands, along with the evaluation of their scavenging activity. The results showed that while complexes **1**, **4**, **6** and **7** did not show any activity, complexes **2**, **3**, **5**, **8** and **9** had moderate antioxidant activity. Complex **2** exhibited significant antioxidant activity, as good and specific activity against superoxide radical, and might be characterized as auspicious SOD-mimics. The results obtained showed that these complexes are good models for the copper–zinc superoxide dismutase, exhibiting the characteristic distorted structure around the copper ion, as well as a marked SOD activity. A positive modulation of this activity though small structural variations could allow to obtain metal complexes that can be potential models for copper sites present in different enzymes.

## Figures and Tables

**Table 1:** IC_50_ values of complexes **1**–**9** towards DPPH and ABTS, and oxygen species radicals.

Sample No.	Compound	DPPH IC_50_ (µM)	ABTS IC_50_ (µM)	Oxygen Species Radicals IC_50_ (µM)			
				O_2_^−^	OH	OOH	NO
**1**	[Cu(Tp^4Bo,5Me^)_2_] 0.5CH_2_Cl_2_	---- ^1^	----	----	----	----	----
**2**	[Cu(Tp^a^)_2_] 0.5H_2_O	----	46.6 ± 3.0	5.6 ± 0.2	----	----	----
**3**	[CuCl_2_(Hpz*^t^*^Bu^)_3_]	76.6 ± 2.5	97.2 ± 3.8	233.0 ± 4.0	----	----	----
**4**	[Zn(Tp^4Bo,5Me^)_2_] CH_2_Cl_2_	----	----	----	----	----	----
**5**	[Zn(Tp^a^)_2_] 4CH_2_Cl_2_	----	64.0 ± 2.5	----	----	----	----
**6**	[Zn(Tp^tBu^)Cl]	----	57.9 ± 3.4	----	----	----	----
**7**	[Cd(Tp^4Bo,5Me^)_2_]	----	----	----	----	----	----
**8**	[Cd(Tp^a^)_2_] 1.5CH_2_Cl_2_	116.0 ± 5.2	----	----	----	----	----
**9**	[Cd(Bp*^t^*^Bu^)(Hpz*^t^*^Bu^)Cl]	----	151.0 ± 4.2	----	----	----	----
**Standard drug**	Trolox	19.9 ± 1.5	4.01 ± 0.7	----	----	----	----

^1^ The empty values are related to a lack of activity of different complexes, also when we tested to a concentration of 5 mM in our experimental conditions.

## Supplementary Materials

The following are available online, Figures S1–S9: FTIR spectra of scorpionate complexes **1**–**9**. Figures S10–S15: ^1^H NMR spectra of diamagnetic complexes **4**–**9**. Figures S16-S18: ^1^H NMR spectra of the free scorpionate ligands.
