# Supplementary material for: Synthesis, Characterization and Assessment of the Antioxidant Activity of Cu(II), Zn(II) and Cd(II) Complexes Derived from Scorpionate Ligands
Source: Molecules. 2020 Nov 13;25(22):5298. doi: 10.3390/molecules25225298 (PMC7696741; doi:10.3390/molecules25225298)

Supplementary information
for the manuscript

Synthesis, characterization and assessment of the antioxidant activity of Cu(II), Zn(II) and Cd(II) complexes derived from scorpionate ligands

Aurel Tăbăcaru*, Rais Ahmad Khan, Giulio Lupidi and Claudio Pettinari*

Figure S1. FTIR spectrum of complex **1**

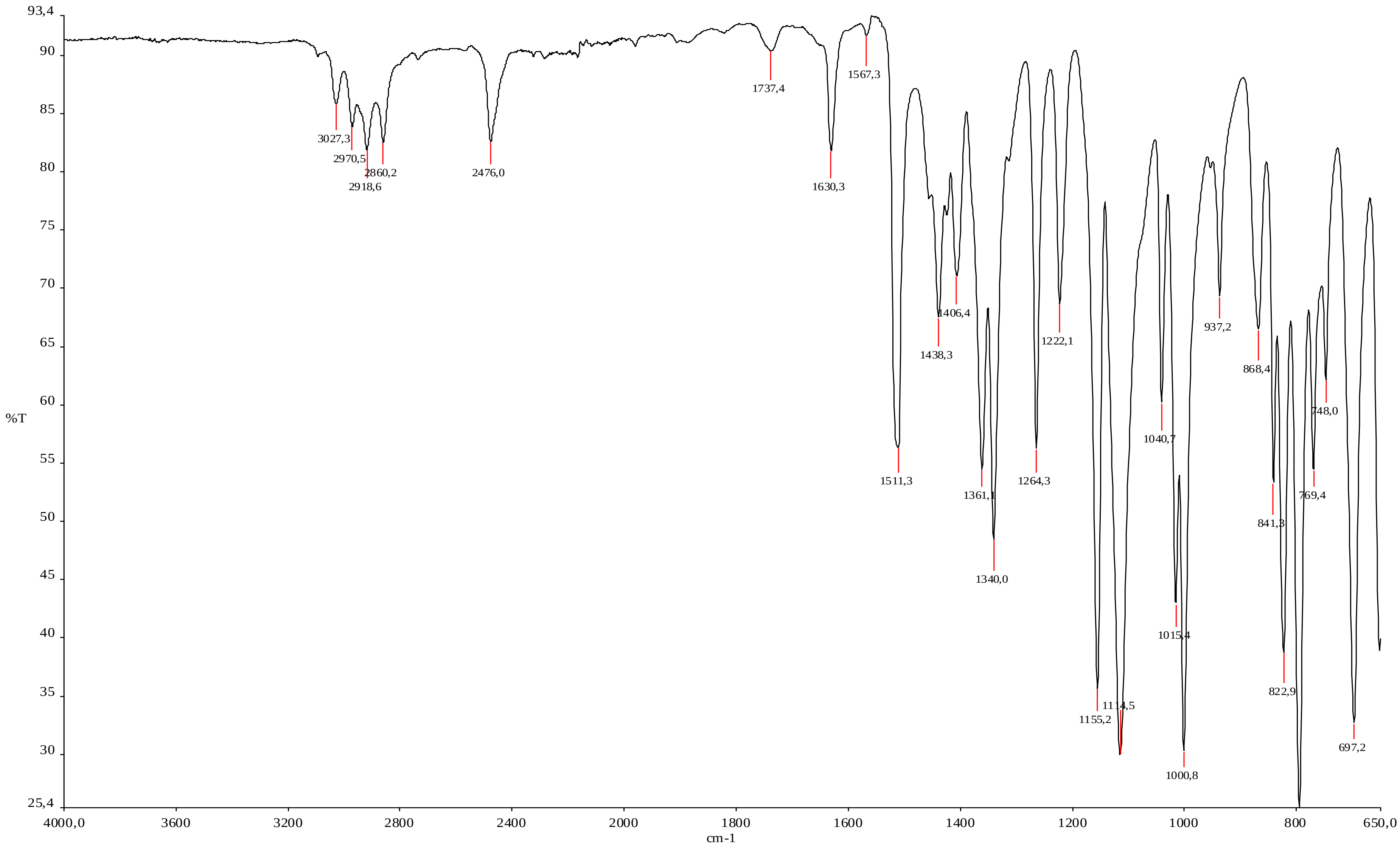

Figure S2. FTIR spectrum of complex **2**

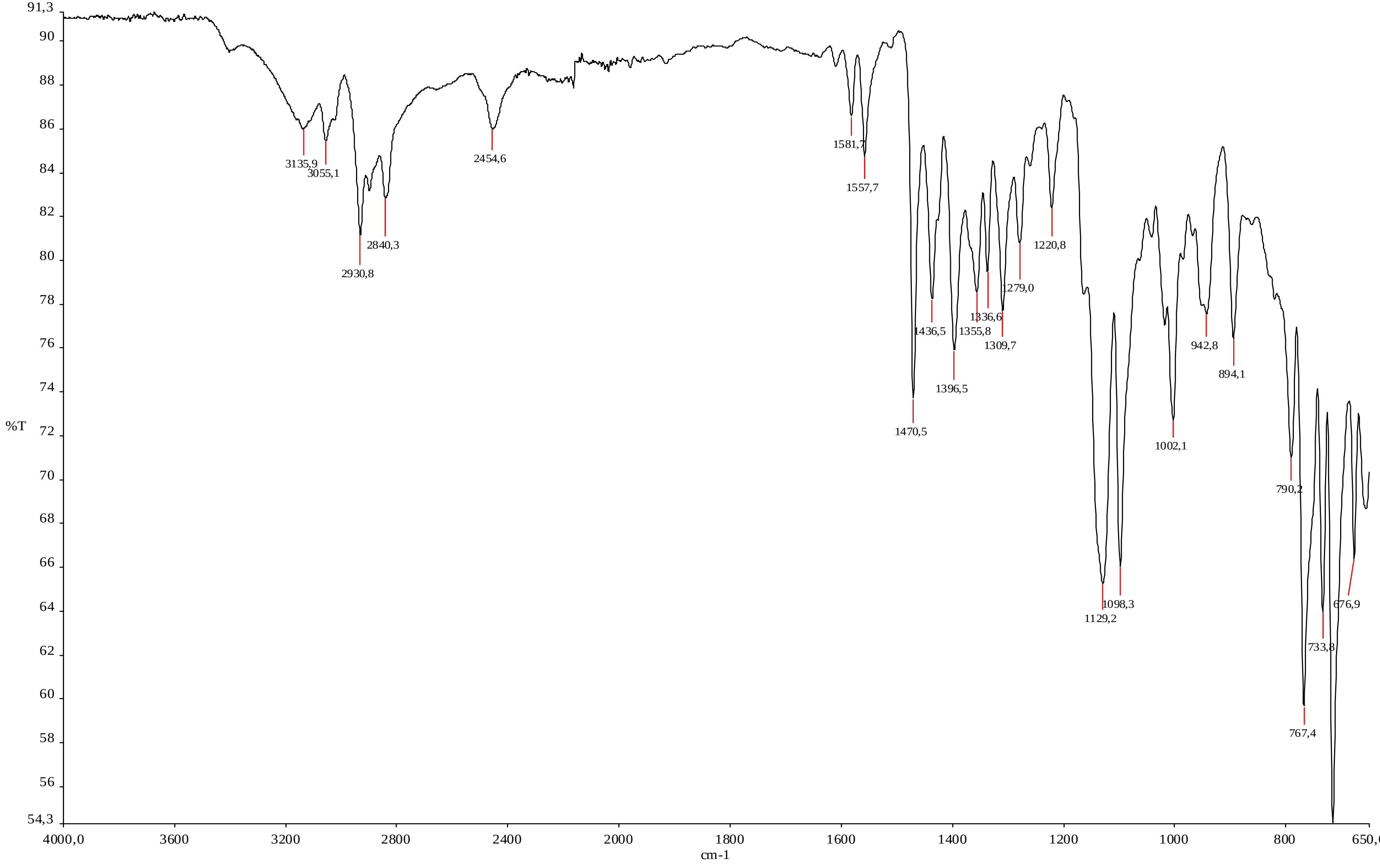

Figure S3. FTIR spectrum of complex **3**

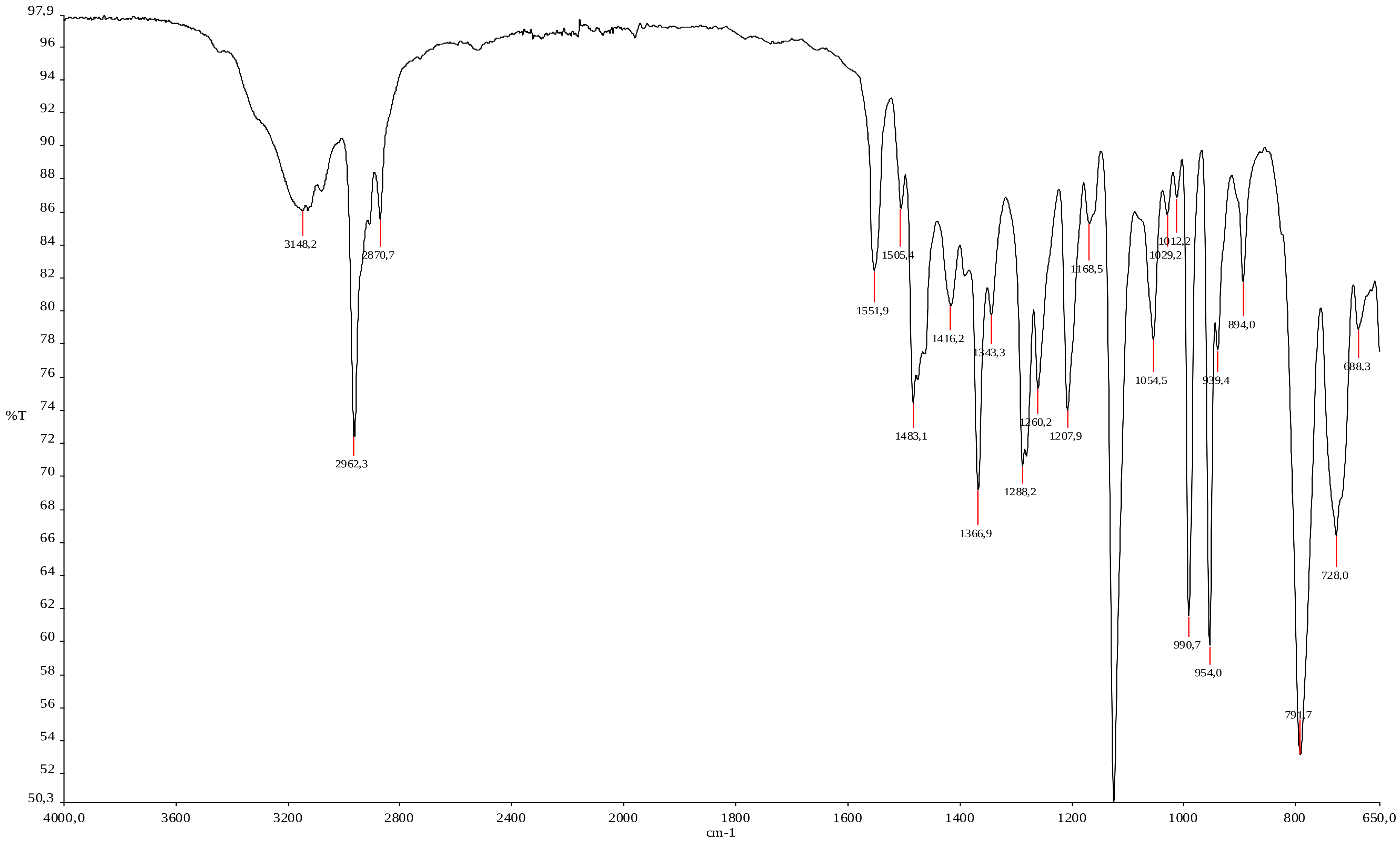

Figure S4. FTIR spectrum of complex **4**

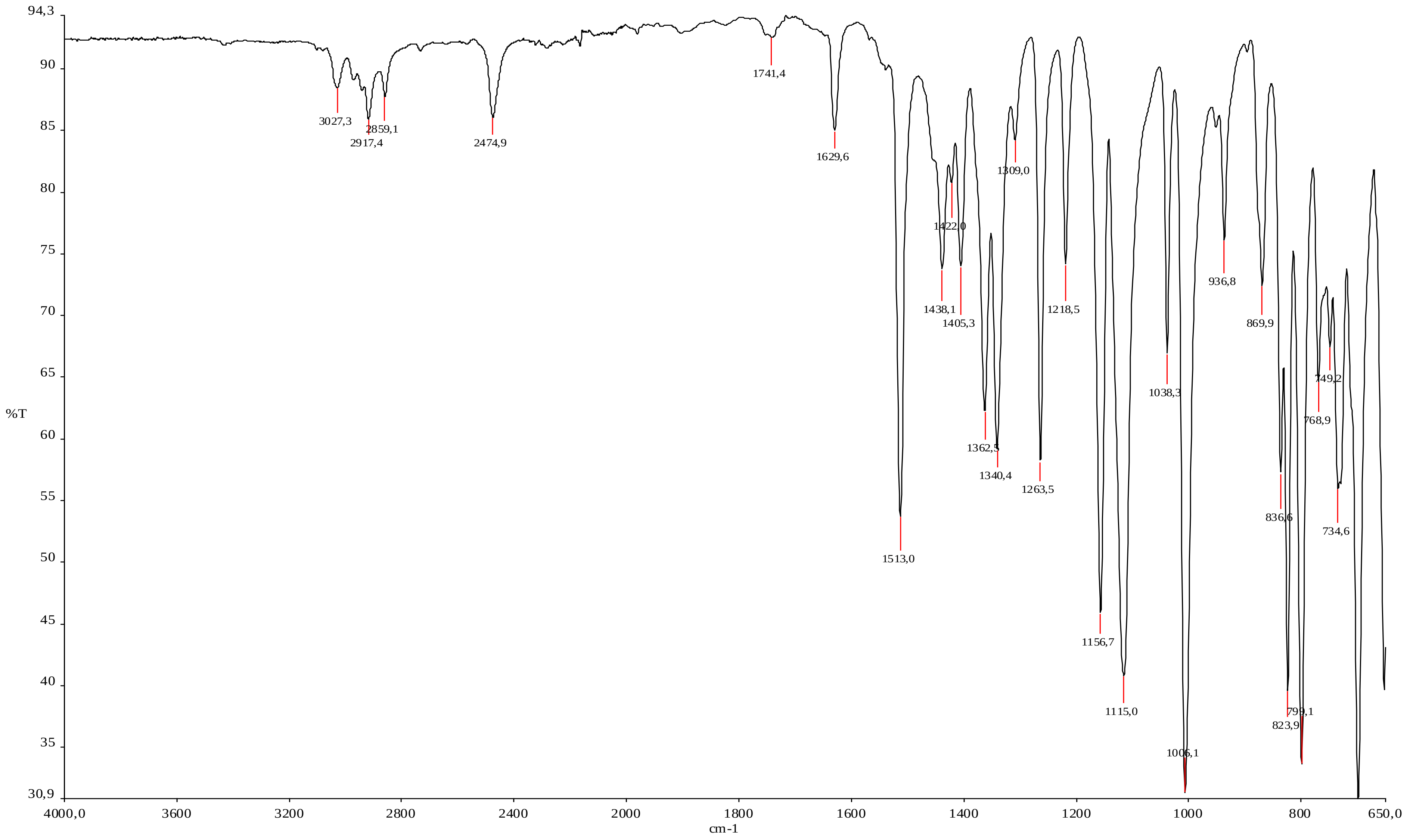

Figure S5. FTIR spectrum of complex **5**

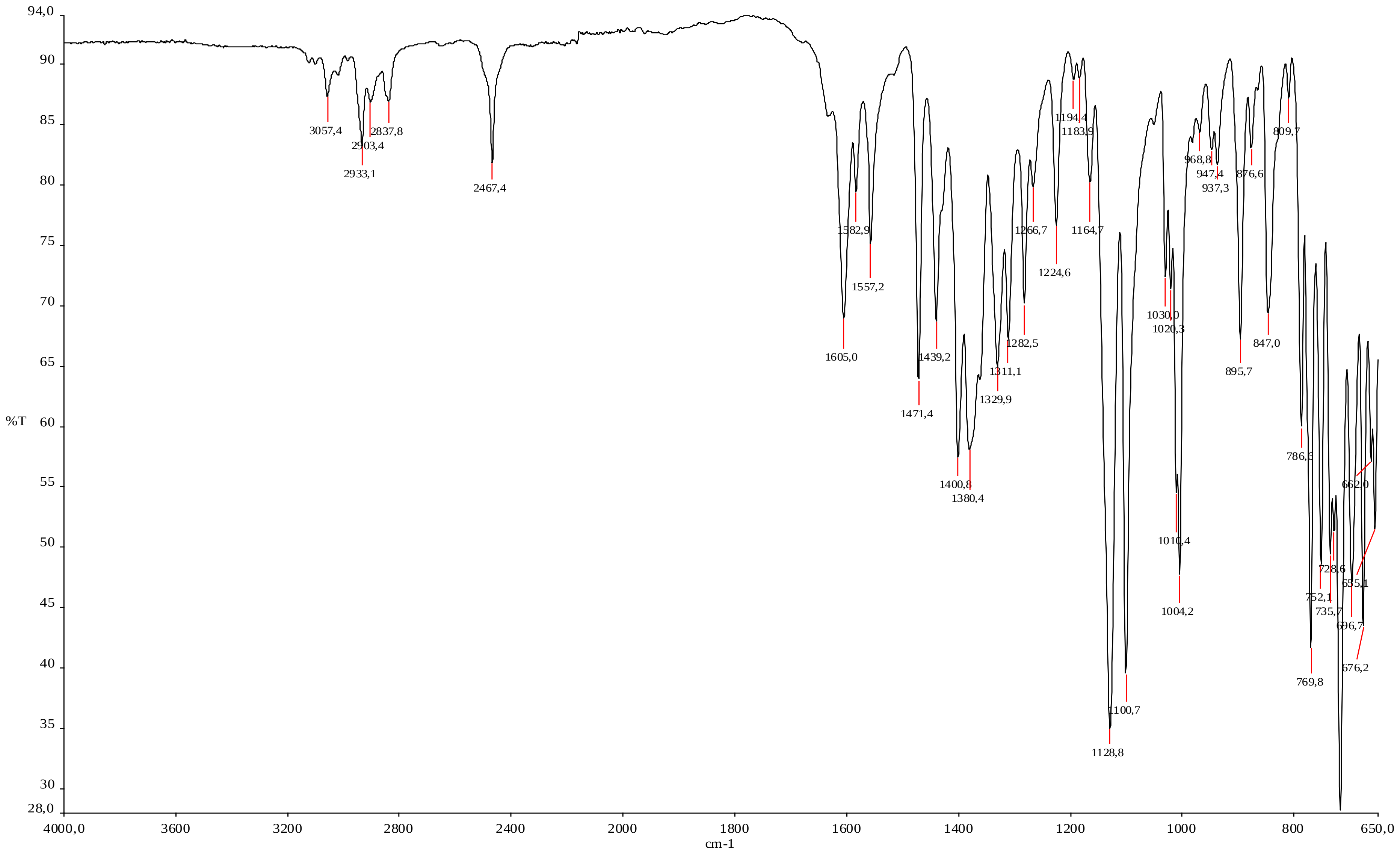

Figure S6. FTIR spectrum of complex **6**

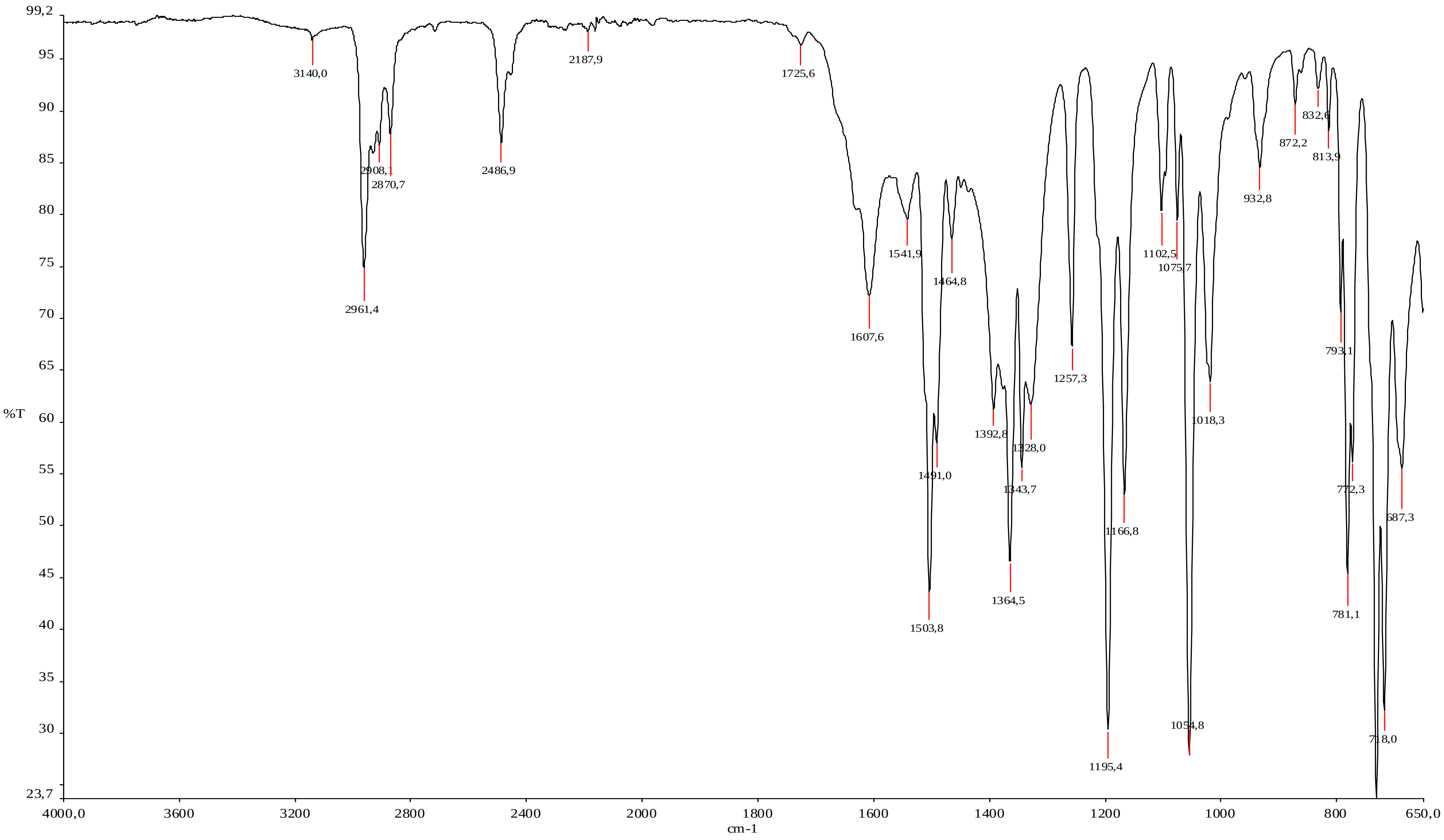

Figure S7. FTIR spectrum of complex **7**

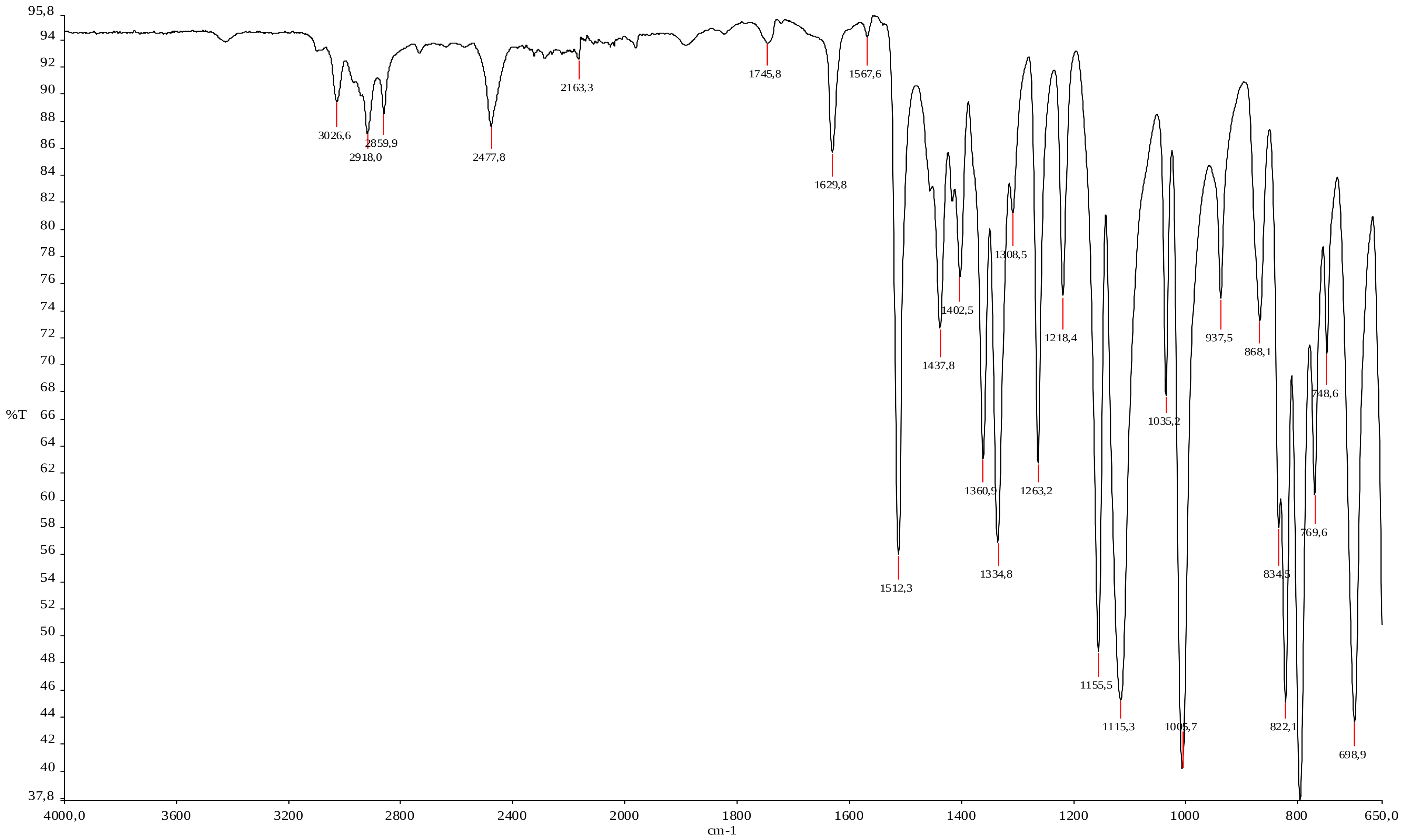

Figure S8. FTIR spectrum of complex **8**

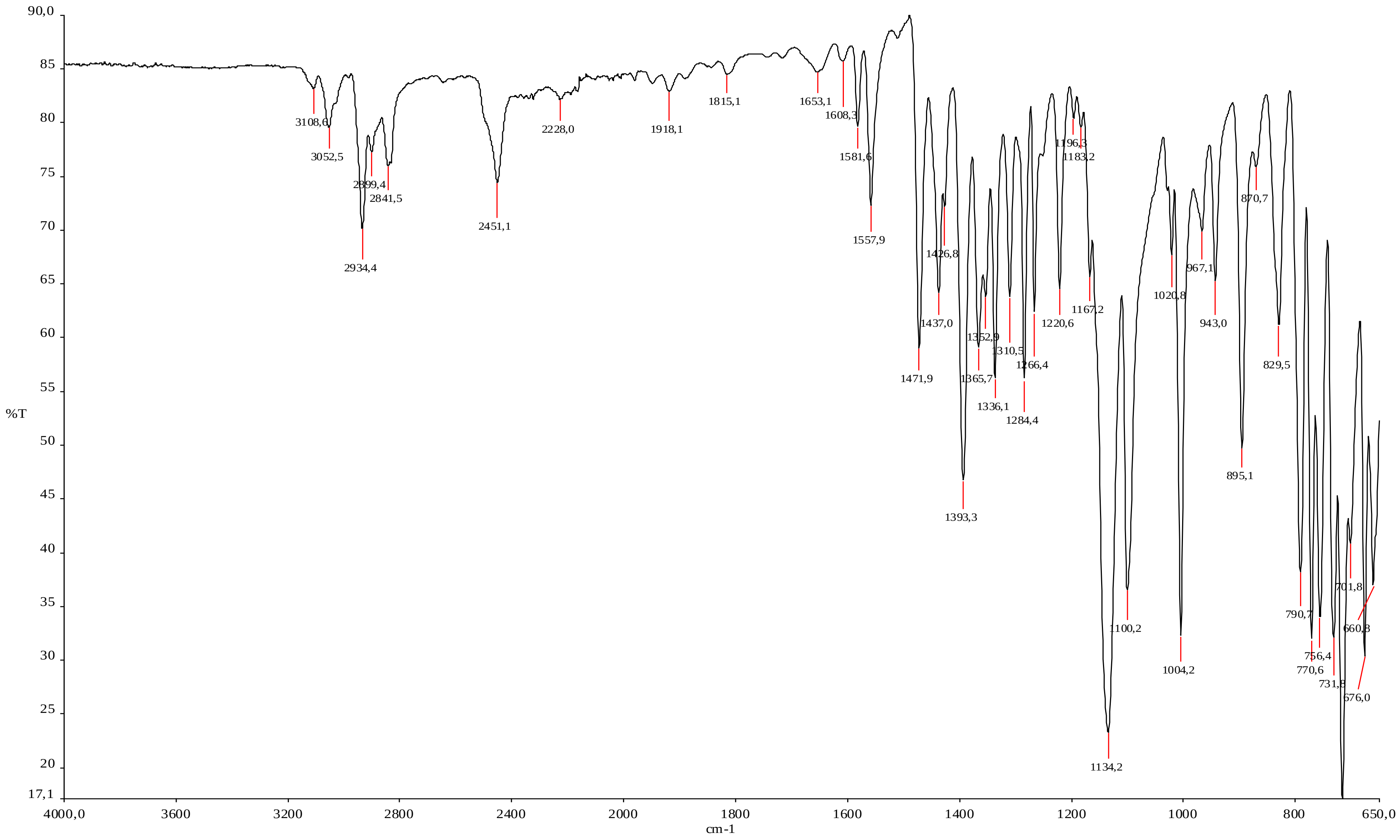

Figure S9. FTIR spectrum of complex **9**

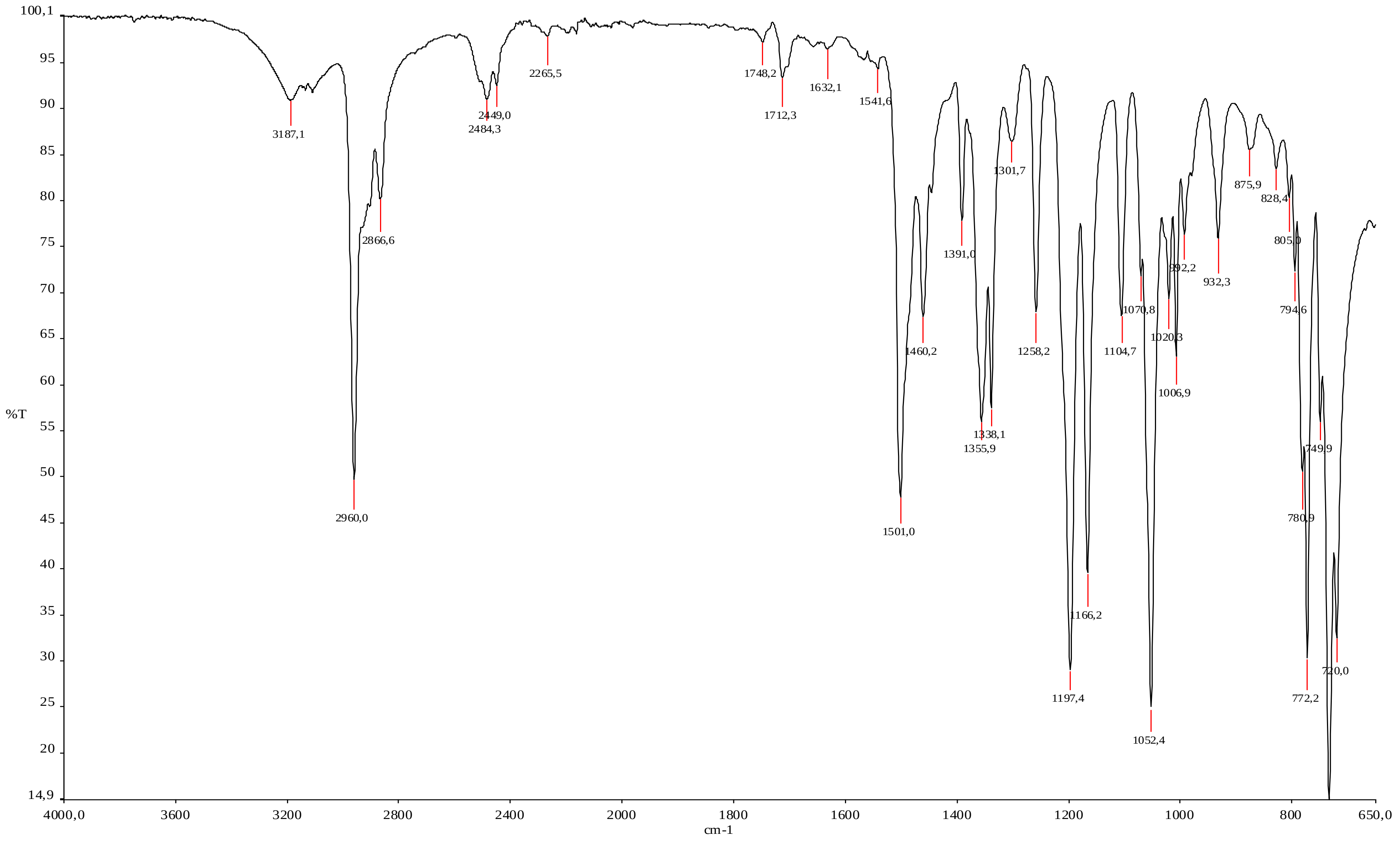

Fig. S10. ^{1}H NMR spectrum of complex **4**

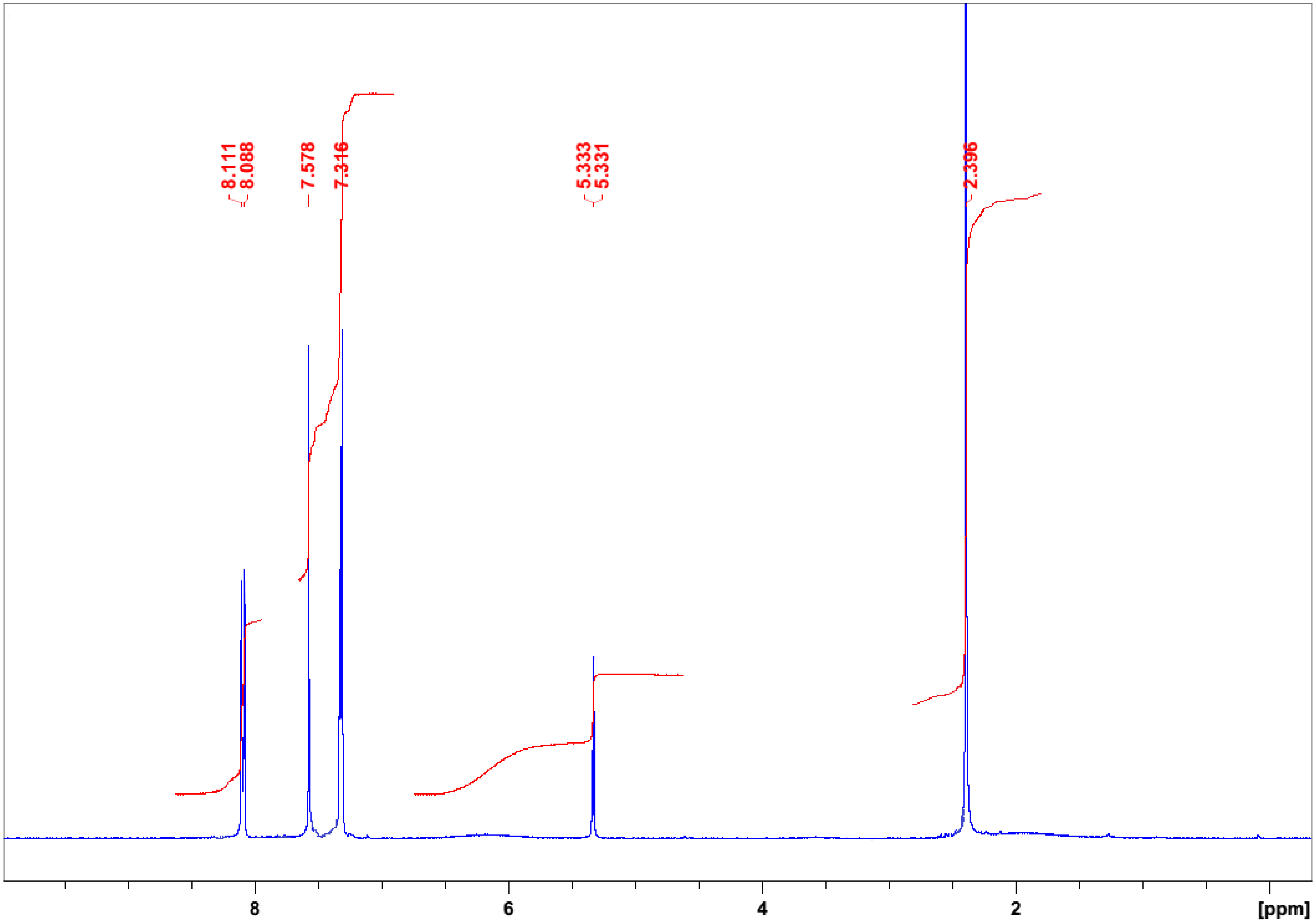

Fig. S11. ^{1}H NMR spectrum of complex **5**

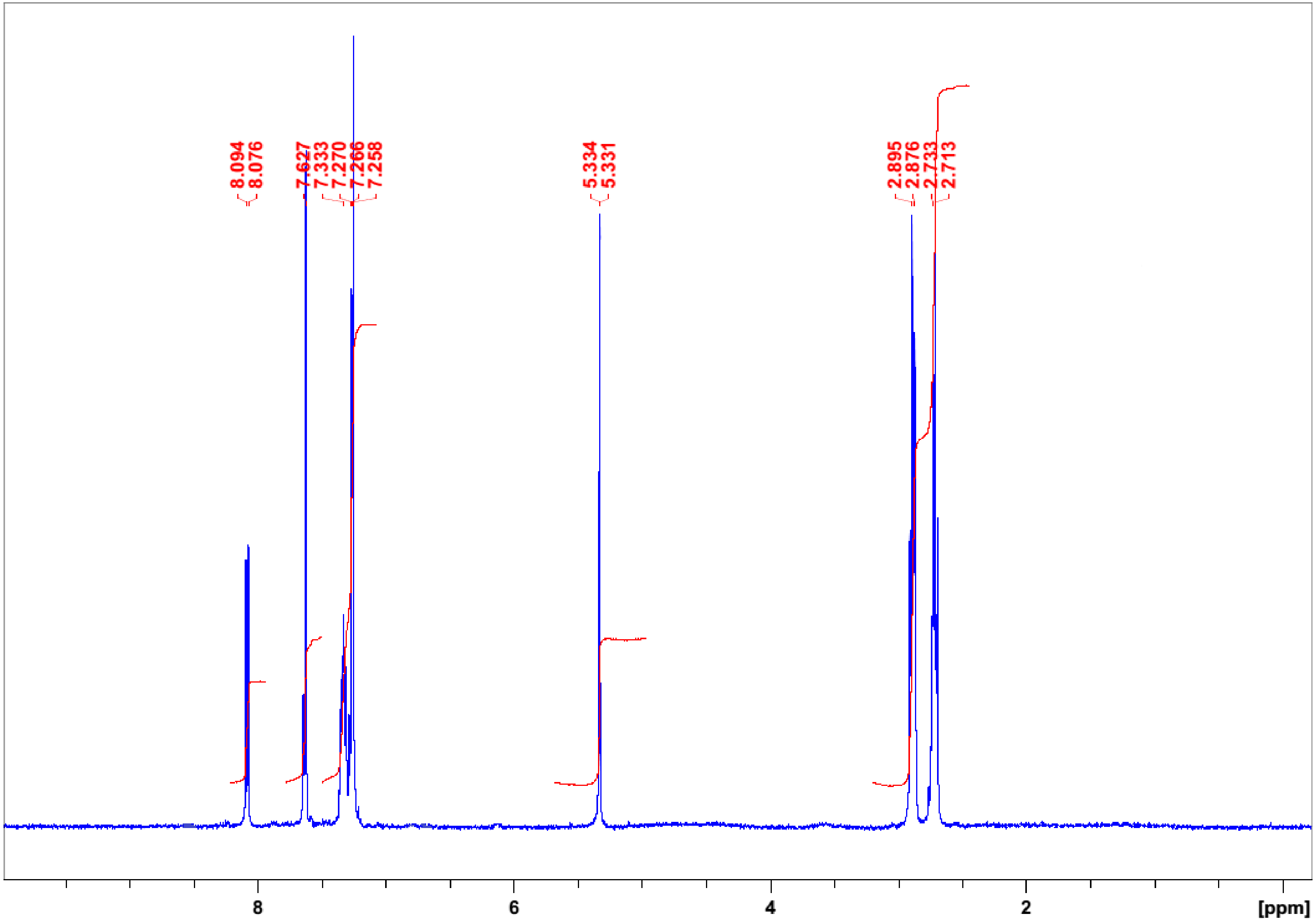

Fig. S12. ^{1}H NMR spectrum of complex **6**

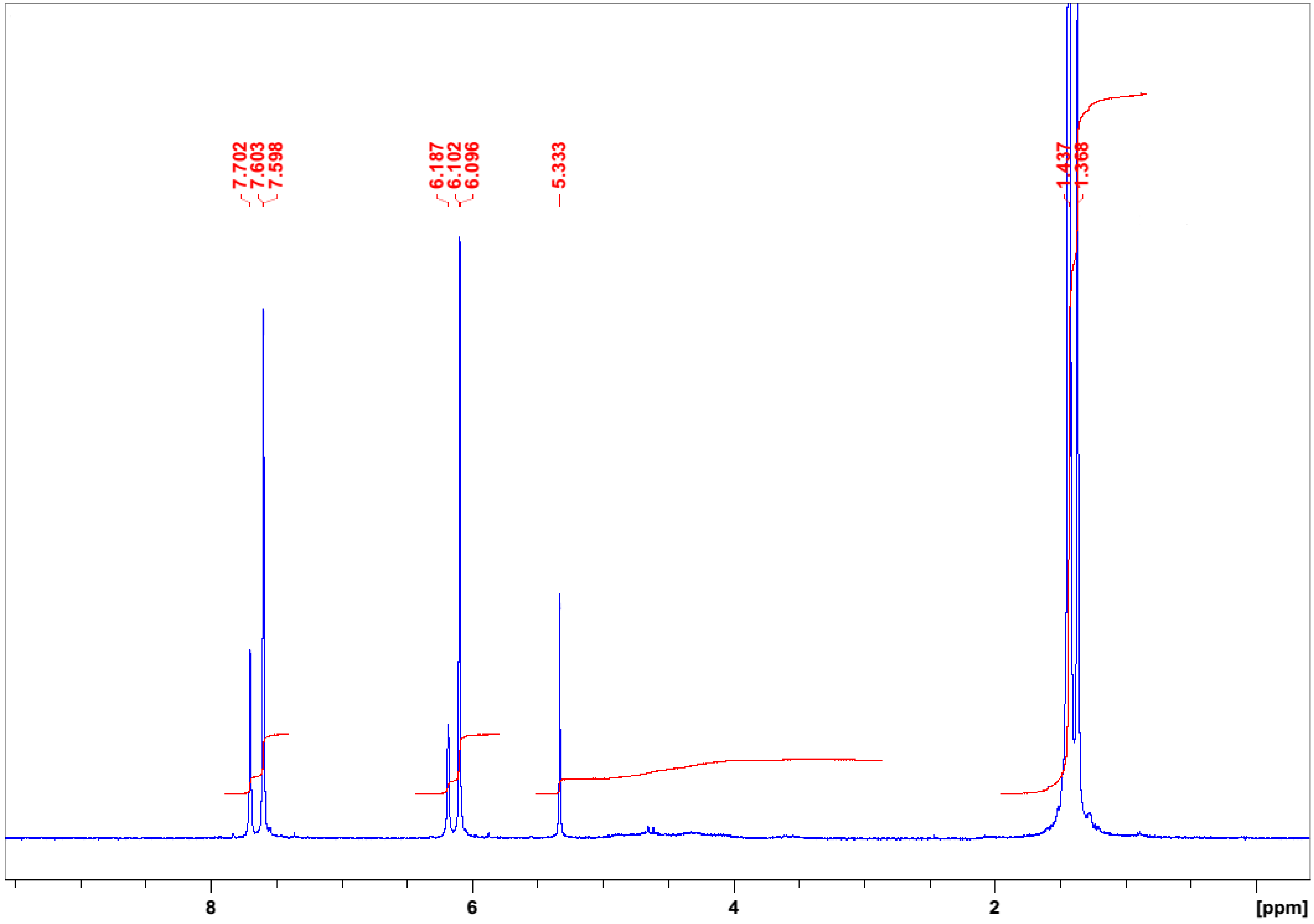

Fig. S13. ^{1}H NMR spectrum of complex **7**

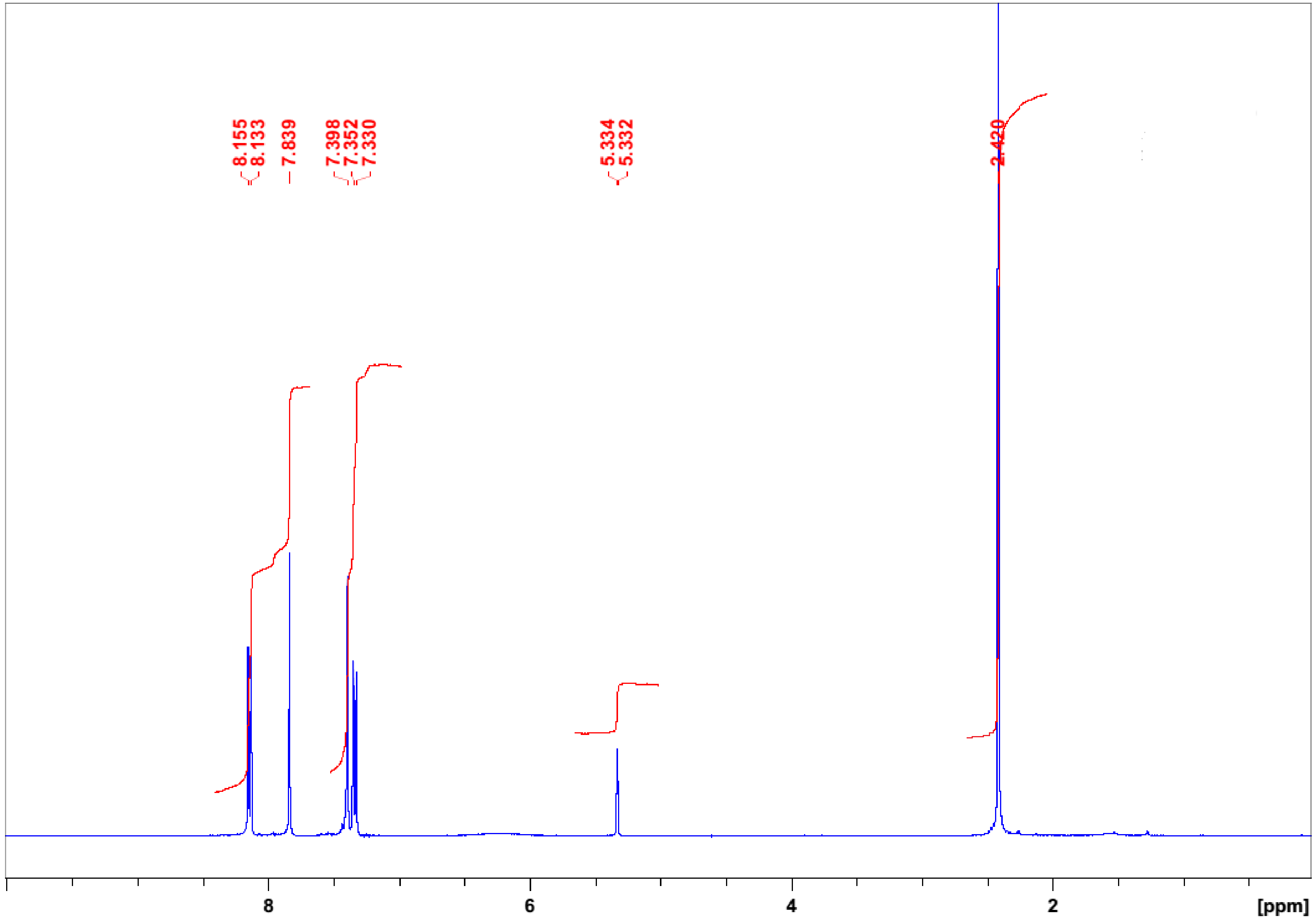

Fig. S14. ^{1}H NMR spectrum of complex **8**

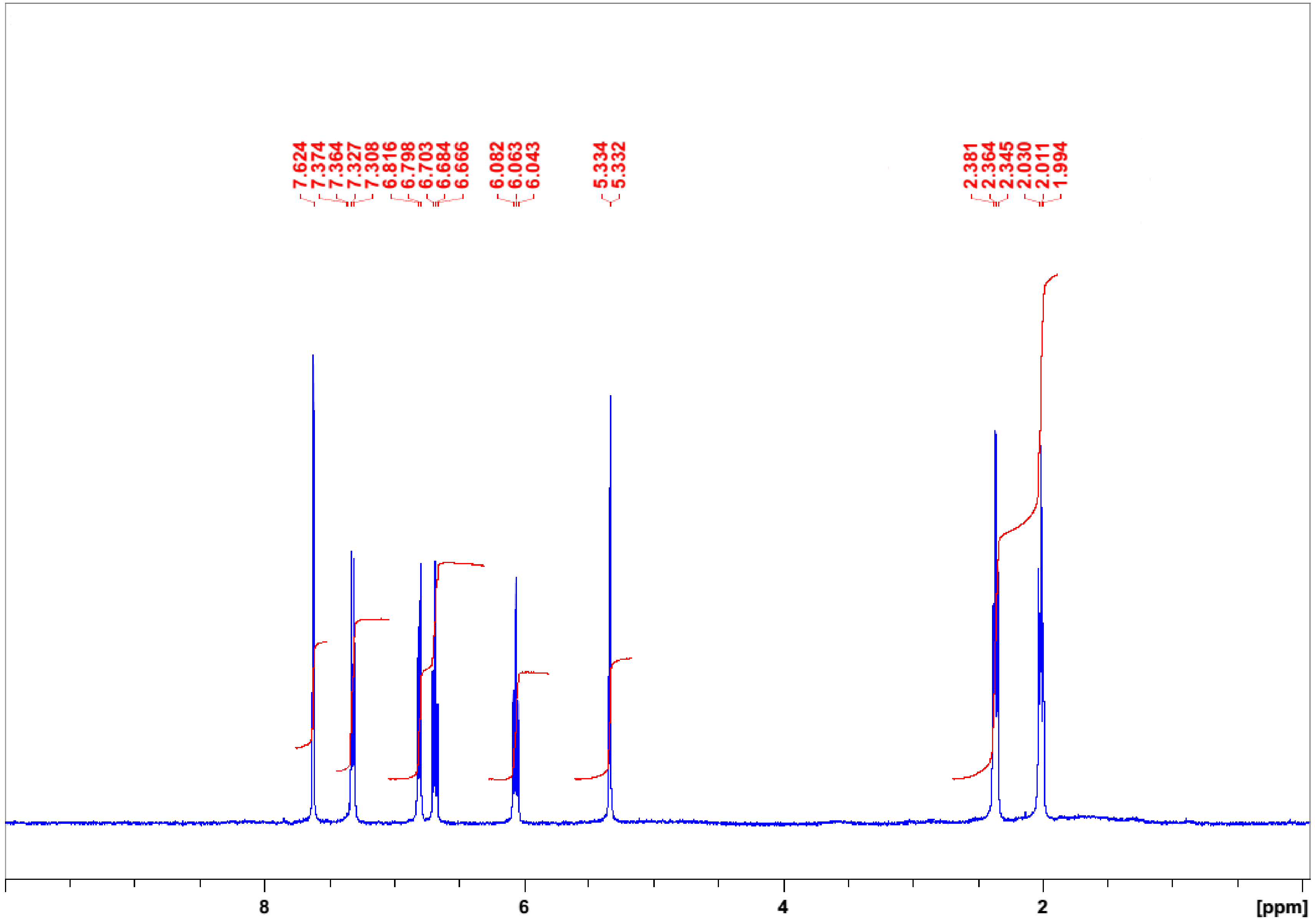

Fig. S15. ^{1}H NMR spectrum of complex **9**

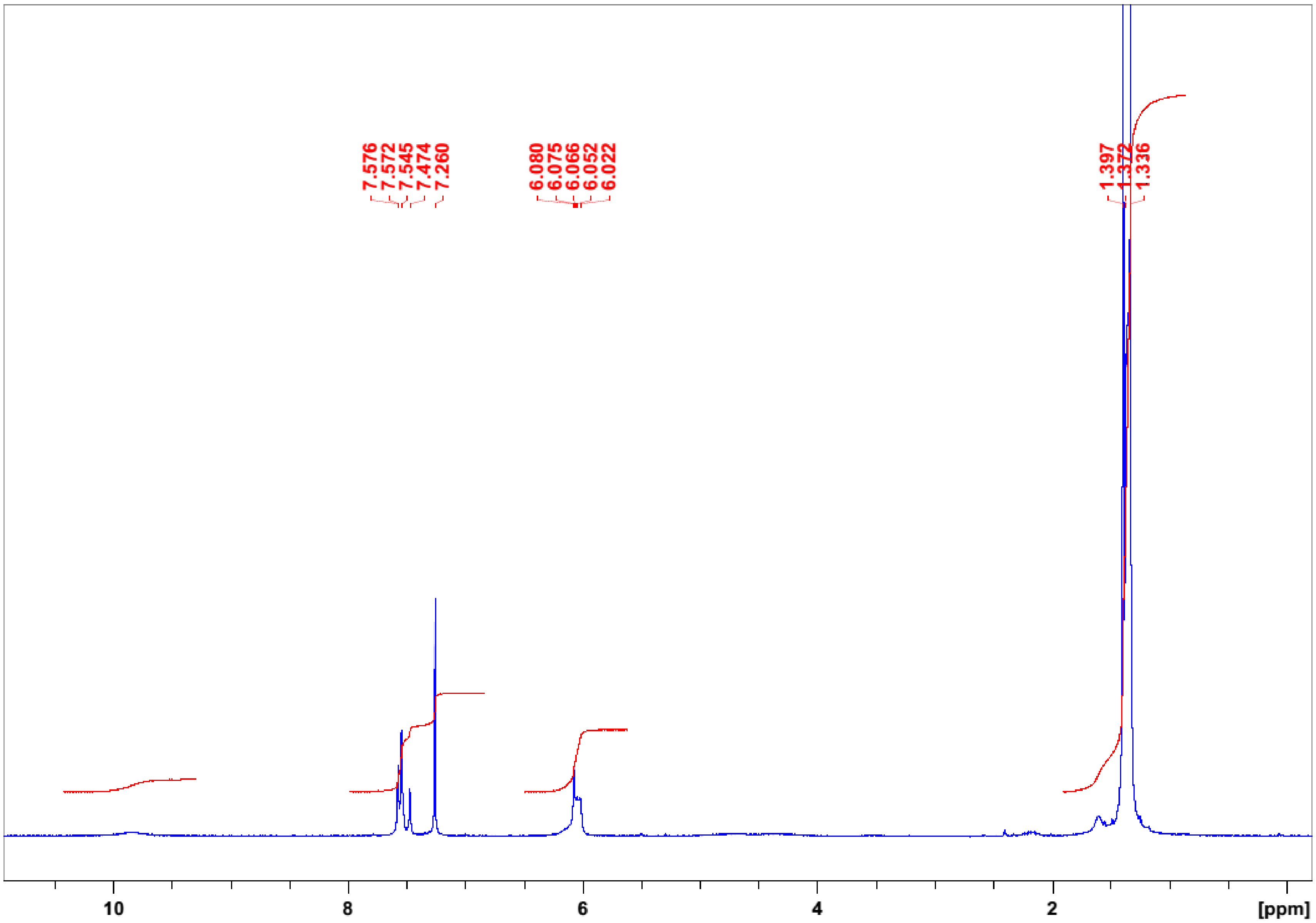

Fig. S16. ^{1}H NMR spectrum of thallium(I) hydrotris(5-methyl-indazolyl)borate ($TlTp^{4Bo,5Me}$)

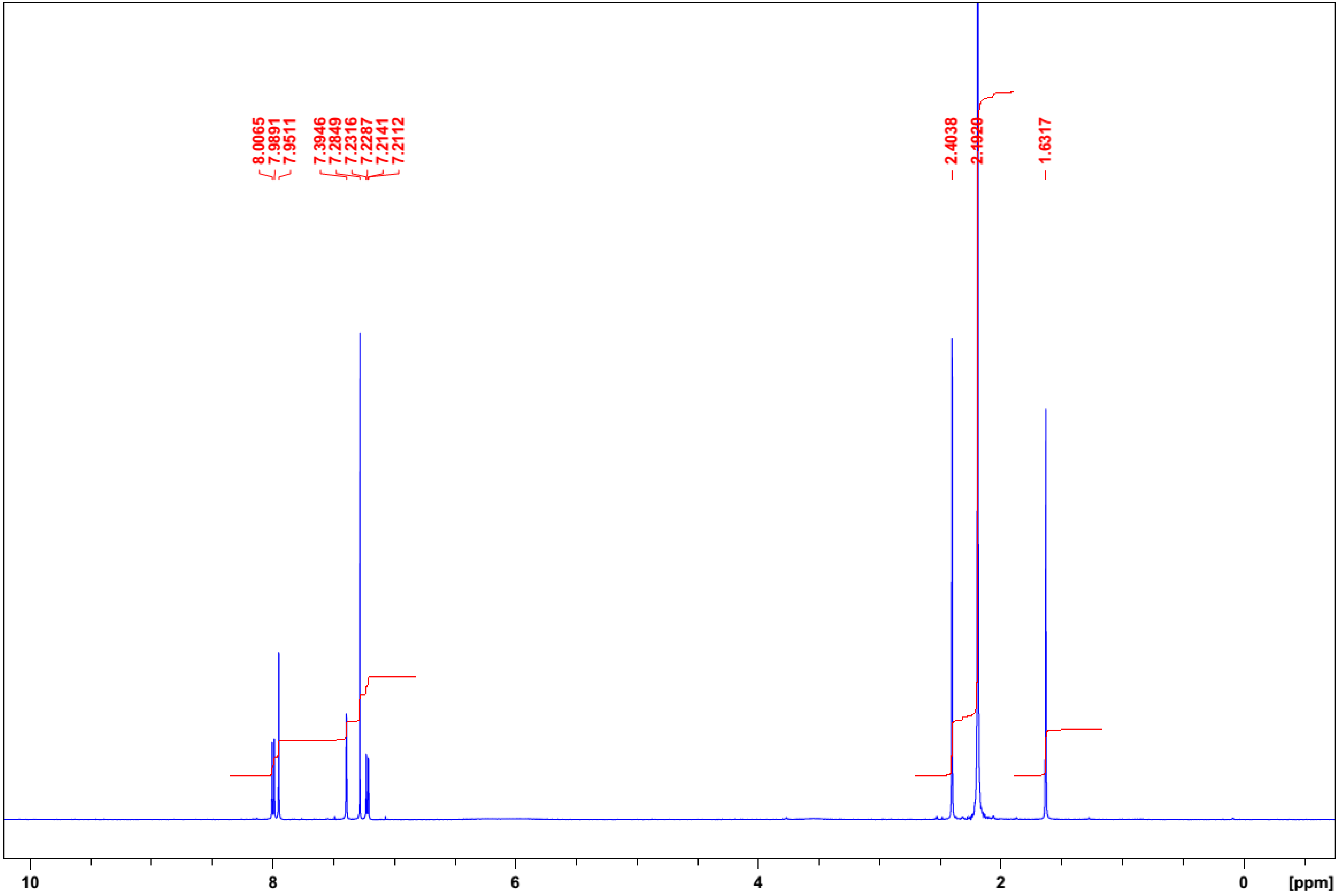

Fig. S17. ^{1}H NMR spectrum of thallium(I) hydrotris(4,5-dihydro-2H-benzo[g]indazolyl)borate (TlTp[a])

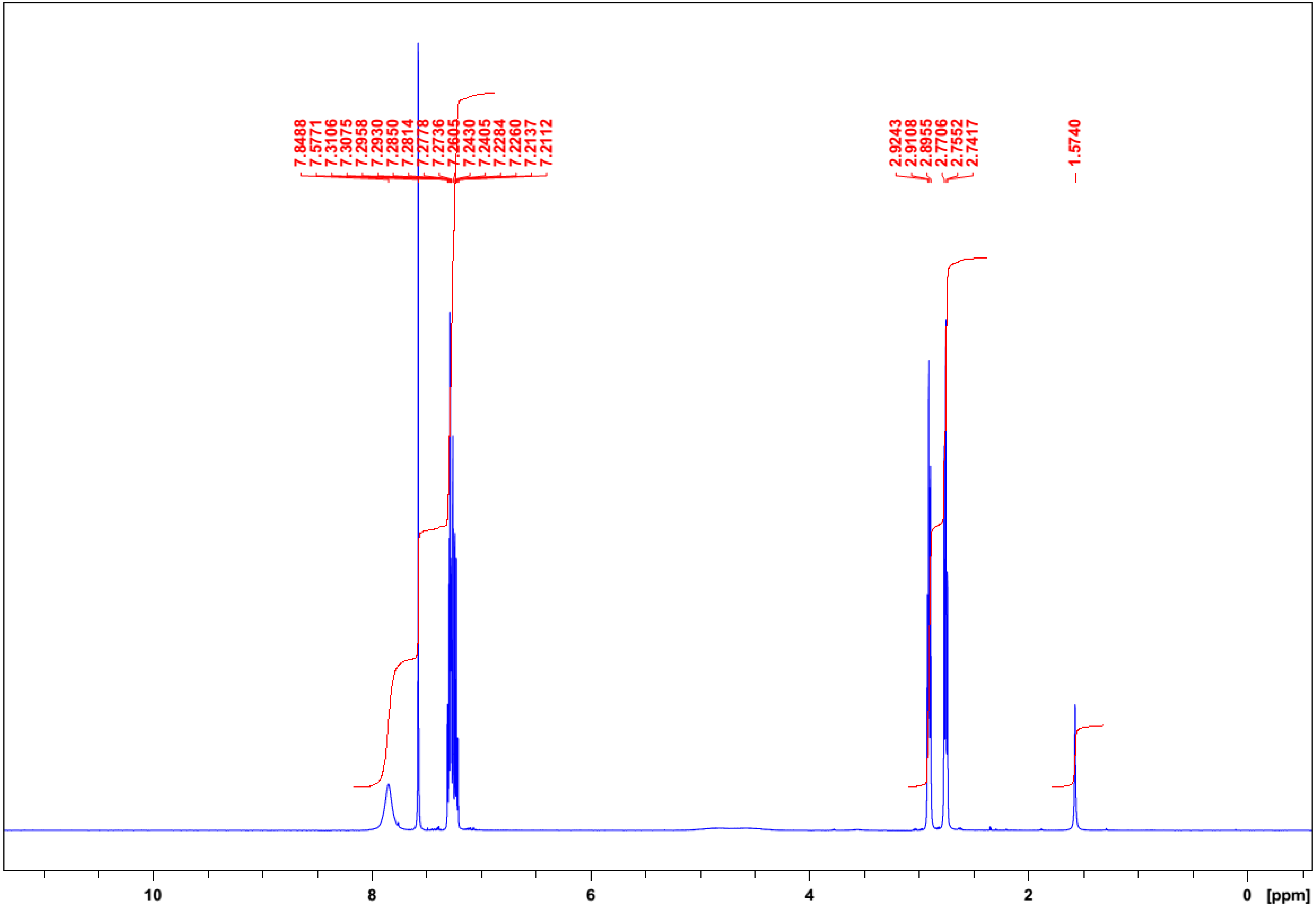

Fig. S18. ¹H NMR spectrum of potassium hydrotris(3-*tert*-butyl- pyrazolyl)borate (KTp^{tBu})

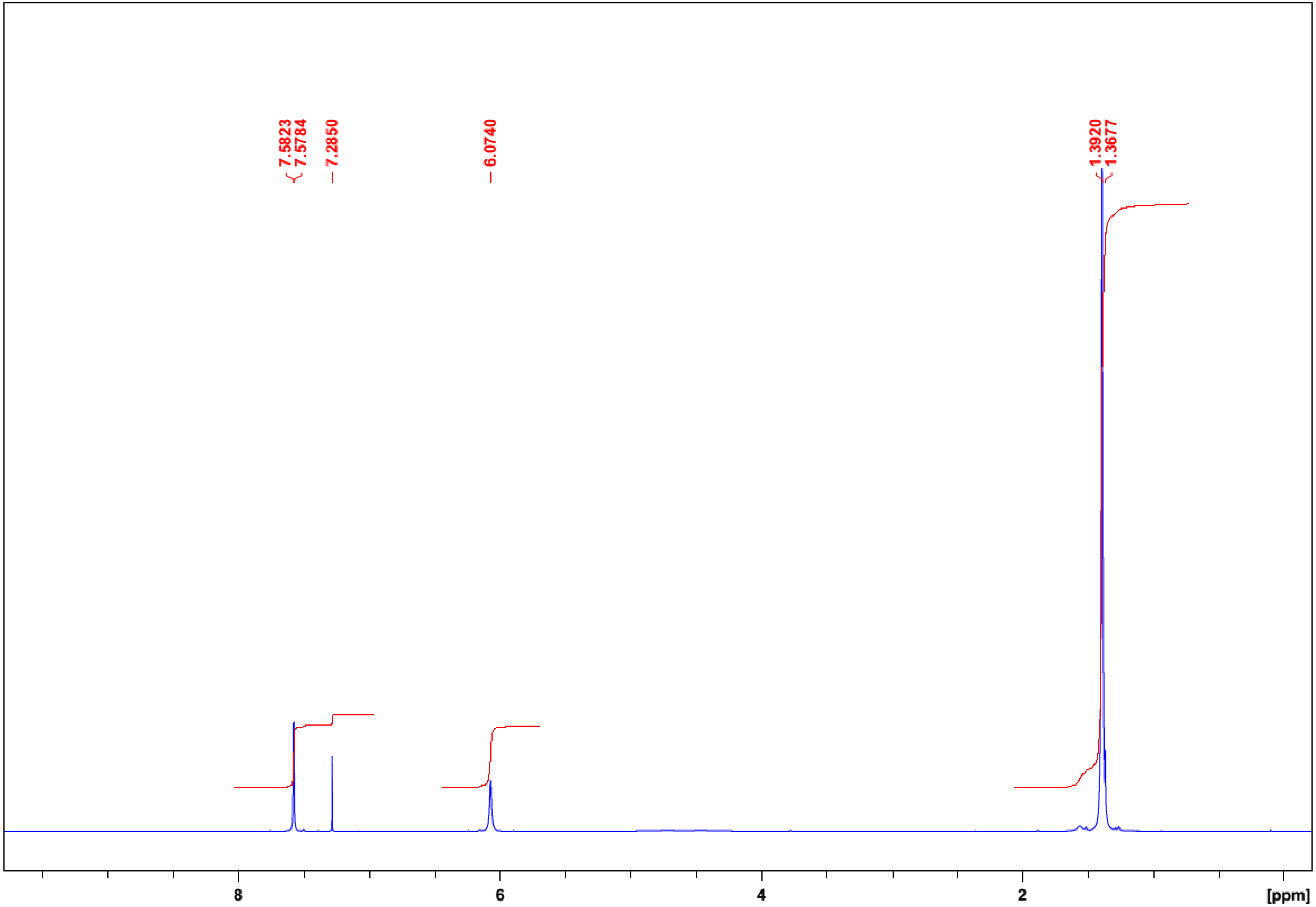

Supplement: Supplementary file 1 [file molecules-25-05298-s001.pdf]
